# Enzymatic strategies for asymmetric synthesis

**DOI:** 10.1039/d1cb00080b

**Published:** 2021-06-01

**Authors:** Mélanie Hall

**Affiliations:** Institute of Chemistry, University of Graz Heinrichstrasse 28 8010 Graz Austria melanie.hall@uni-graz.at; Field of Excellence BioHealth – University of Graz Austria

## Abstract

Enzymes, at the turn of the 21st century, are gaining a momentum. Especially in the field of synthetic organic chemistry, a broad variety of biocatalysts are being applied in an increasing number of processes running at up to industrial scale. In addition to the advantages of employing enzymes under environmentally friendly reaction conditions, synthetic chemists are recognizing the value of enzymes connected to the exquisite selectivity of these natural (or engineered) catalysts. The use of hydrolases in enantioselective protocols paved the way to the application of enzymes in asymmetric synthesis, in particular in the context of biocatalytic (dynamic) kinetic resolutions. After two decades of impressive development, the field is now mature to propose a panel of catalytically diverse enzymes for (i) stereoselective reactions with prochiral compounds, such as double bond reduction and bond forming reactions, (ii) formal enantioselective replacement of one of two enantiotopic groups of prochiral substrates, as well as (iii) atroposelective reactions with noncentrally chiral compounds. In this review, the major enzymatic strategies broadly applicable in the asymmetric synthesis of optically pure chiral compounds are presented, with a focus on the reactions developed within the past decade.

## Abstract 2

Asymmetric synthesis achieved with enzymes for stereoselective reduction and bond forming reactions, enantioselective and atroposelective reactions.

## Graphical abstract

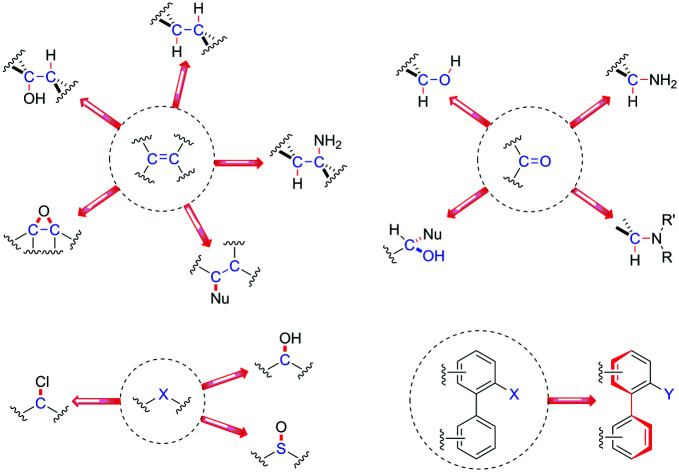

## Introduction

Biocatalysis, which can be broadly defined as the use of enzymes to catalyze chemical reactions, is found in diverse fields of application. While its historical use by ancient civilizations in brewery, bakery and wine making was not conscious, advances mostly in the 20th century in neighboring disciplines such as biochemistry, enzymology, molecular biology, and more recently in protein crystallography, genetic engineering and computational tools, contributed to a global and molecular understanding of enzymes and their modes of action. At the same time, scientists could finally produce, manipulate and modify enzymes on demand, which participated in the establishment of modern biocatalysis.^1,2^ The use of enzymes in chemistry has a long tradition, such as in the food, textile and detergent industry,^3,4^ however biocatalysis started to dramatically impact the fine chemical and pharmaceutical industry only recently at the turn of the 21st century. Besides the strong motivation to develop environmentally more acceptable chemical processes, the faster access to proteins and the ability to modify them largely contributed to a mindset shift in industry.^5,6^ Enzymes can compete with traditional chemical (catalytic) methods for a broad range of chemical transformations, including those long considered nonnatural. Exemplary is the identification of the catalytic activity of aldoxime dehydratases in the Kemp elimination reaction^7^ – a base-catalyzed deprotonation of a benzisoxazole ring – for which, besides chemical bases, only *de novo* designed proteins were known to be active catalysts.^8^

Baeyer–Villiger monooxygenases (BMVOs) represent another beautiful example of how nature designs efficient enzymatic machineries to catalyze difficult chemical reactions. Chemists employ peracids or peroxides as oxidant in the eponymic reaction for the insertion of oxygen between two carbon atoms. After attack of the carbonyl group of the substrate by the oxidant, the reaction proceeds through formation of the so-called Criegee intermediate. The collapse of this intermediate leads to migration of one of the alkyl substituents of the substrate carbonyl to the oxygen atom derived from the oxidant, and eventually results in the formation of the ester or lactone product. BVMOs simply use molecular oxygen as oxidant in the enzymatic version of the reaction and rely on a reduced flavin cofactor to activate the oxygen. The resulting peroxyflavin is a powerful nucleophile that can attack carbonyls, leading to a biological Criegee intermediate. The rest of the reaction can be considered analogous to the chemical oxidation.^9^ A major advantage of the biological catalyst is the benign and nontoxic environment required for the oxygenation reaction: air, importantly seconded by high regio- and enantioselectivity.

The interest in using enzymes in asymmetric synthesis is motivated by the exquisite chemo, regio- and stereoselectivity of enzymes. Concepts underlying enzymatic strategies in asymmetric reactions are similar to those found in organic synthesis:

– A prochiral compound can be transformed stereoselectively into a chiral product in enantiopure form. Usually, such reactions involve nucleophilic attack onto sp^2^ hybridized carbon atoms embedded in a C=C-, C=O-, or C=N-double bond, and imply face recognition of the substrate molecular plane by the enzymes.

– A prochiral sp^3^ hybridized atom can be converted into a chiral center by the formal enantioselective replacement of one of the two enantiotopic groups, such as hydrogen atoms on carbon or electron lone pairs on sulfur.

– In case the chiral information is already present, a racemic substrate can be converted enantioselectively according to the principles of kinetic resolution.

– Finally, the case of atroposelective reactions deserves attention. The chiral information is contained not in a center but in an axis that can be either formed or, if already existing, resolved.

In this article, the major strategies relying on enzymes for the key step of asymmetric synthesis are reviewed and classified in one of the following categories: (i) stereoselective reactions involving transformations of sp^2^ hybridized carbon atoms, (ii) enantioselective reactions and (iii) atroposelective reactions. For each category, the focus was intentionally put on most recent literature where relevant, while each strategy is introduced in the light of the underlying chemical concept.

Nonnatural asymmetric reactions catalyzed by P450 monooxygenases and pioneered by Frances Arnold are out of the scope of this review. Excellent and comprehensive articles can be recommended for the interested reader.^10–14^ The enzymes selected for this review were chosen due to a certain level of generality and broad applicability in synthesis, usually indicated by a large substrate scope. In selected cases, (industrial) examples that largely contributed to the acceptance of biocatalysis in asymmetric synthesis^15^ are presented in details. The broad topic covering the use of hydrolases in enantioselective reactions was not included,^16–18^ except on special occasions. Finally, special techniques leading to optically pure chiral compounds, such as desymmetrization, stereoinversion, cyclic deracemization and enantioconvergent processes^19–26^ were intentionally not broadly covered.

## 1. Stereoselective reactions involving transformations of sp^2^ carbons

**Scheme 1:**
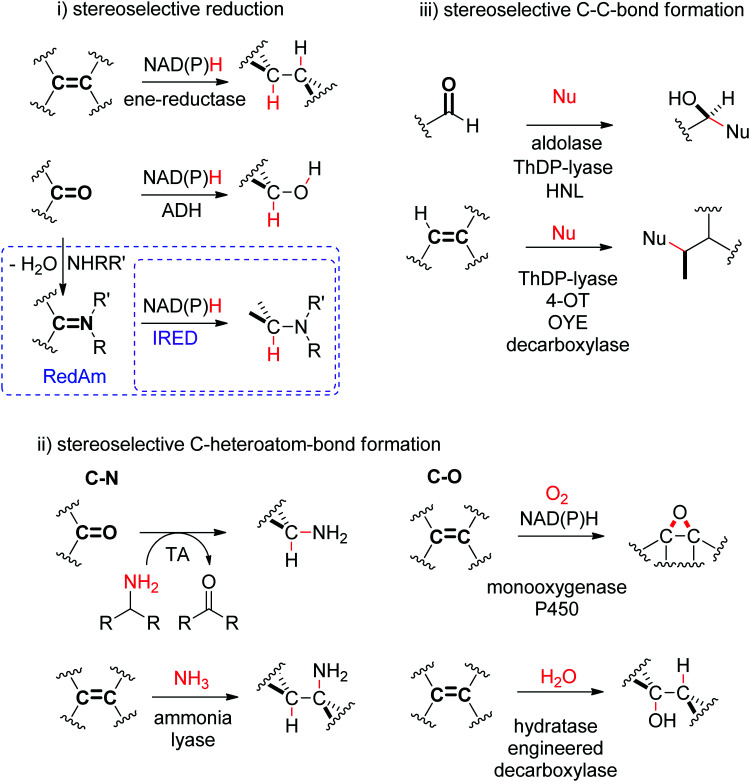
Enzymatic asymmetric strategies relying on stereoselective reactions: (i) reduction, (ii) C–heteroatom-bond formation and (iii) C–C-bond formation by addition of nucleophilic carbon (4-OT: 4-oxalocrotonate tautomerase, ADH: alcohol dehydrogenase, HNL: hydroxynitrile lyase, IRED: imine reductase, OYE: Old Yellow Enzyme, RedAm: reductive aminase, TA: transaminase, ThDP: thiamine diphosphate, Nu: nucleophile).

The biocatalytic stereoselective conversion of prochiral molecules into chiral products often involves substrates bearing at least one sp^2^ hybridized carbon atom embedded in a double bond, such as C=C-, C=N- or C=O-bond. Transformations of these double bonds include reduction reactions (stereoselective C–H bond formation), transamination and hydroamination (stereoselective C–N-bond formation), epoxidation (stereoselective C–O-bond formation), and addition of nucleophilic carbon (stereoselective C–C-bond formation). All these reactions proceed through selective substrate face recognition by the enzyme of interest and may lead to the formation of up to two chiral centers (Scheme 1).

### 1.1. C=C-, C=O-, C=N-double bond reduction

In biology, the reduction of double bonds is typically a formal addition of [2H], which proceeds *via* a hydride addition/protonation sequence. The hydride added stereoselectively on the most electron-deficient (carbon) atom is derived from a molecule of nicotinamide cofactor NAD(P)H,^27^ and usually, the final product is obtained by protonation – often catalyzed by an acidic amino acid – through the reaction medium.

#### 1.1.1. Alkene reduction

Several types of enzymes are active in the stereoselective reduction of C=C-double bonds.^28^ Ene-reductases from the flavoprotein family of Old Yellow Enzymes (OYEs) have become the golden standard for this reaction in biocatalysis owing to a broad substrate scope and large protein diversity.^29,30^ Bacterial enoate reductases from obligate anaerobes are specific for α,β-unsaturated carboxylic acids (enoates). Due to their dependence on an [Fe–S]-cluster, these enzymes are oxygen-sensitive and therefore less suitable for practical applications in synthesis. Nicotinamide-dependent reductases from the NADP-dependent leukotriene B4 dehydrogenase subfamily of medium chain dehydrogenases/reductases (MDR) are flavin-independent enzymes found in plants and mammals, which have not yet demonstrated generality through high stereoselectivity on a large panel of substrates. Some of these alkene reductases have however displayed sufficient stereoselectivity and could be applied in the synthesis of a few chiral molecules.^31–33^

**Scheme 2:**
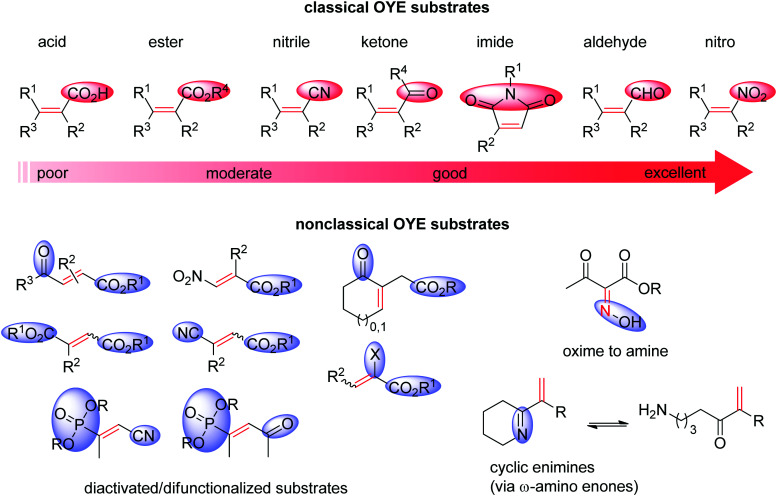
Classical substrates for Old Yellow Enzymes (OYEs) listed according to reactivity, and nonclassical substrates.

**Scheme 3:**
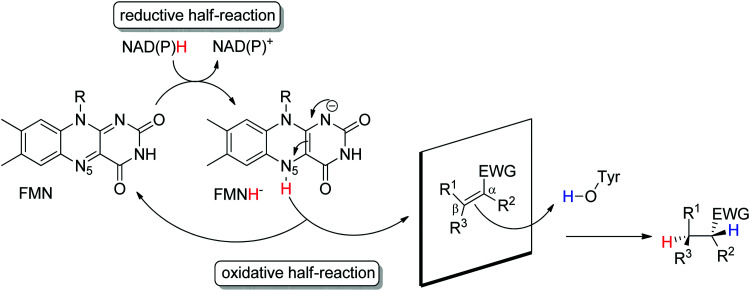
Stereoselective reduction of activated alkenes by FMN-dependent Old Yellow Enzymes (EWG: electron-withdrawing group, FMN: flavin mononucleotide).

Ene-reductases from the flavin mononucleotide (FMN)-dependent OYE family^34^ accept a broad range of α,β-unsaturated compounds activated on Cα by an electron-withdrawing group, and that include nitro, carbonyl, nitrile, imide and carboxylic acid derivatives (Scheme 2).^30^ Strongly activated oximes can also be reduced.^35^ Based on detailed structural and mechanistic studies, the reaction is well understood and proceeds according to a ping-pong bi–bi-mechanism. In a first step (the reductive half-reaction), FMN is reduced by NAD(P)H. Following exit of the oxidized cofactor from the active site, binding of the activated substrate by two conserved residues (His/His or His/Asn) *via* H-bonding with the electron-withdrawing group further contributes to reduce the electron density of the C=C-double bond and favors attack by the reduced flavin. The transfer of the hydride from N_5_ onto Cβ (the oxidative half-reaction) is followed by protonation at Cα from a conserved Tyr residue located on the other side of the C=C-double bond, ensuring overall *trans*-specific addition of 2H (Scheme 3).^36,37^ This is in contrast to the *cis*-specific homogeneous hydrogenation methods. In case of mixed binding modes^38^ of the substrate, the stereoselectivity may be affected and the ee values of the product become moderate to poor.^39–41^ Homologues have been discovered in all kingdoms of life and many crystal structures of OYEs are now available, thereby fueling protein engineering campaigns toward improved catalytic properties.^42,43^

**Fig. 1:**
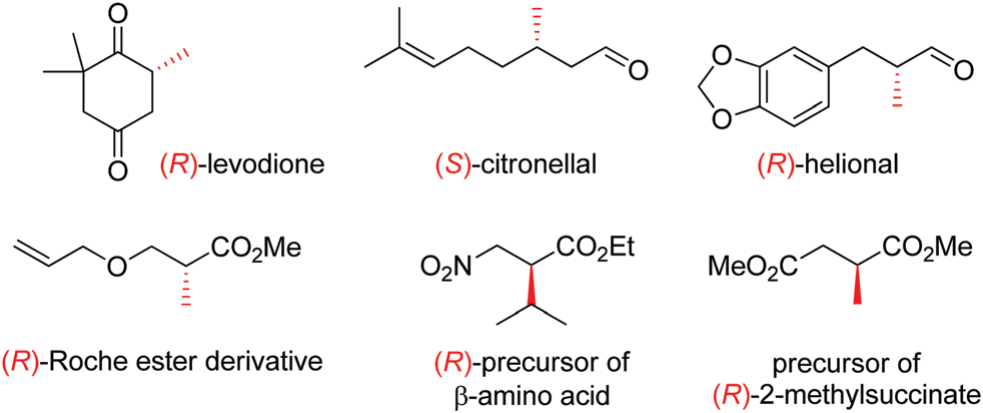
Important chiral building blocks obtained by stereoselective bioreduction catalyzed by ene-reductases of the OYE family.^44–50^

OYEs have been exploited in a number of reactions in order to generate chiral molecules in enantiopure form, many of which find application as small building blocks in synthesis. For instance, (*R*)-levodione,^44^ (*S*)-citronellal^45,46^ and (*R*)-helional^47^ were obtained by OYE-catalyzed reduction reactions. Similarly, this biocatalytic platform was employed to access (*R*)-derivatives of the Roche ester,^48^ the (*R*)-nitro precursor of a β-amino acid^49^ and the precursor of (*R*)-2-methylsuccinate (Fig. 1).^50^

**Scheme 4:**
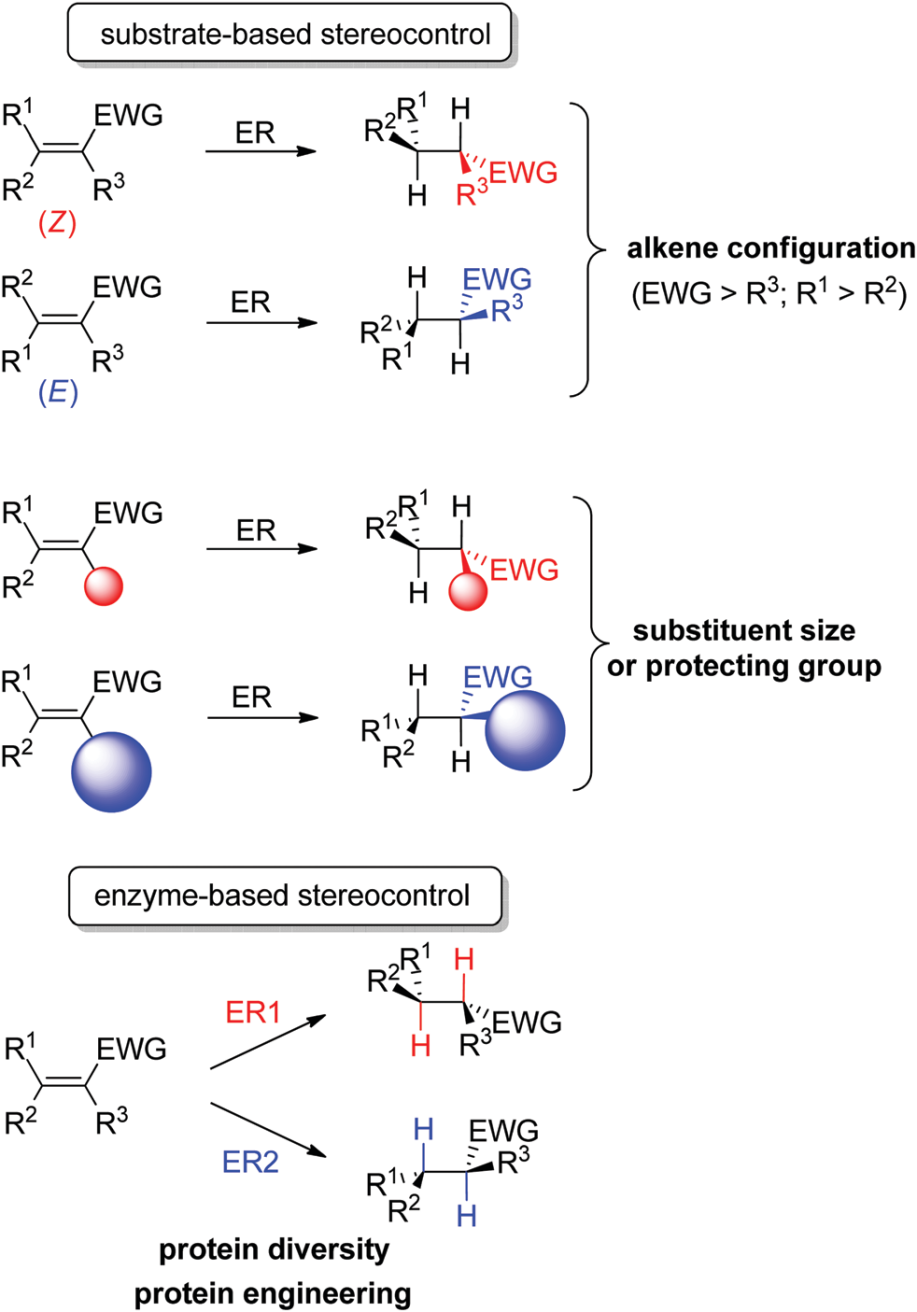
Stereocontrol strategies with ene-reductases based on substrate or enzyme (ER: ene-reductase, EWG: electron-withdrawing group).

An important advantage of this biocatalytic reductive platform is the availability of two main strategies to control the stereochemical outcome of the reactions^51–56^ (Scheme 4): (i) in substrate-based methods, modulation of the alkene configuration or the type and size of the substituents impacts the absolute configuration of the product. (ii) In enzyme-based methods, advantage is taken of the large protein diversity – and the resulting variation in enzyme active site configuration – to identify stereocomplementary enzymes. In addition, protein engineering can be employed to modify on purpose the active site toward altered substrate binding and thus influence the stereoselectivity of the reaction.

**Scheme 5:**
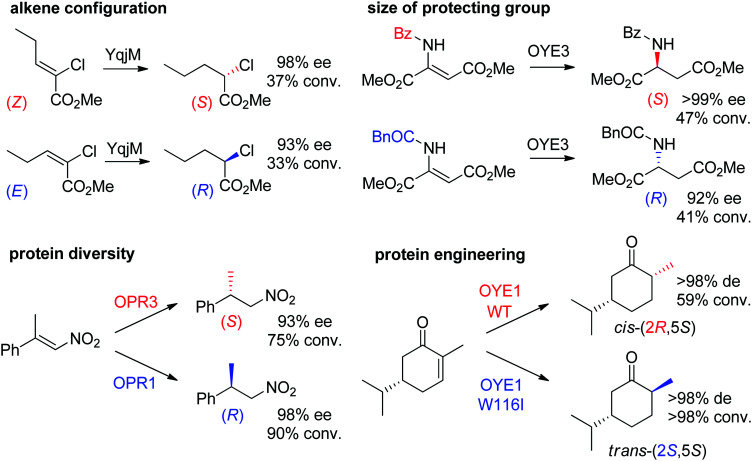
Examples of stereocontrol strategies using ene-reductases.^42,45,57,58^ Reprinted from ref. 252 Copyright 2020, with permission from Elsevier.

Some examples can be highlighted (Scheme 5): the use of YqjM to access both enantiomers of methyl 2-chloropentanoate by using either (*Z*)- or (*E*)-configurated methyl 2-chloropent-2-enoate as starting material;^57^ the conversion by OYE3 of α,β-dehydroamino acids bearing a benzoyl- or phenylacetyl-protected amino group into the (*S*)- or (*R*)-product, respectively;^58^ the use of either OPR1 or OPR3, both isoenzymes from *Lycopersicon esculentum*, to generate (*R*)- or (*S*)-1-nitro-2-phenyl-propane, respectively;^45^ the reduction of (*S*)-carvone to the *cis*-(2*R*,5*S*)- or *trans*-(2*S*,5*S*)-dihydrocarvone product by wild-type OYE1 or its variant W116I, respectively.^42^ Both stereocontrol strategies can be efficiently merged, as was shown in the reduction of cyclohexenone substrates bearing an enol ether moiety in the α-position toward the corresponding acyloin derivatives. The pair methoxy group/OYE3 led to the formation of the (*R*)-enantiomer in 97% ee while the pair benzyloxy/XenB yielded the (*S*)-enantiomer with 96% ee.^59^

**Scheme 6:**
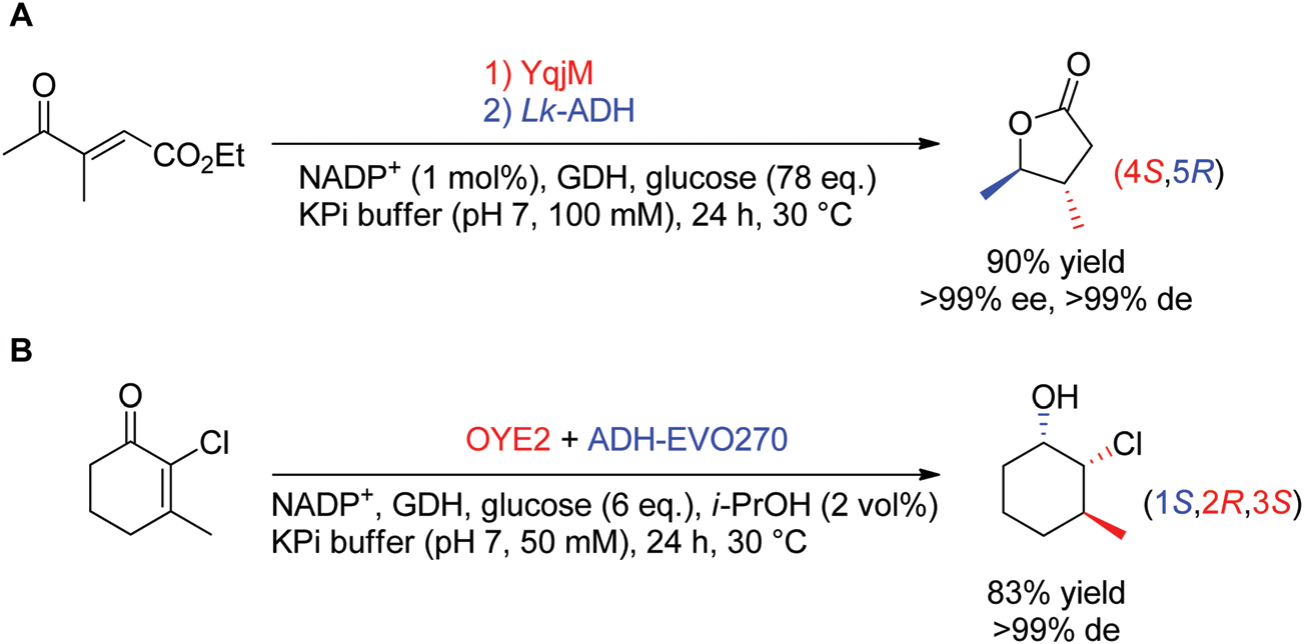
Access to two (A) or three (B) chiral centers *via* bi-enzymatic reductive cascades in (A) one-pot two-step^60^ and (B) one-pot one-step.^61^ A recycling system was used in both cases to regenerate NADPH (ADH-EVO270: commercial alcohol dehydrogenase, GDH: glucose dehydrogenase, *Lk*-ADH: ADH from *Lactobacillus kefir*).

In theory, the stereoselective reduction of tetrasubstituted alkenes by OYEs can generate up to two chiral centers. In practice, such highly substituted compounds are challenging to obtain in highly optically pure form, often due to the racemization of the labile α-center and the steric constraints posed by the substrate substitution in the enzyme active site. In a few published examples, the corresponding products were obtained with high diastereoselectivity (Scheme 6).^60,61^ In both reported cases, the resulting products were further reduced by alcohol dehydrogenases, thereby granting access to γ-butyrolactones with two, and halohydrins with three contiguous stereocenters, respectively, with excellent ee and de values (>99%).

Further recent developments in the field^62^ include reductive (dynamic) kinetic resolution of racemic substituted lactones through the stereorecognition of a distant γ-chiral center,^63^ and reduction of chemically rich molecules (with more than one functional group). In the latter case, several difunctionalized products could be obtained and reduction was particularly favored in the case of substrates undergoing double activation (Scheme 2).^41,49,58,64^

Despite the large spectrum of applications in synthesis at the laboratory scale, ene-reductases from the OYE family are still underrepresented in industrial biocatalytic processes.^50^ As a consequence of the enzyme typically low substrate concentration tolerance (<20 mM), efforts in process engineering are required to render OYE-catalyzed reactions compatible with large scale process conditions. Immobilization of ene-reductases may increase process robustness. Lately, co-immobilization with a cofactor recycling enzyme partner showed encouraging results.^65–68^

Even more challenging remains the difficult predictability in the substrate acceptance and stereopreference, and currently, identification of a biocatalyst suitable for a desired reaction is inevitably tied up with large screening efforts.

Lately, ene-reductases could be successfully employed in the C=C-bond reduction of cyclic enimines (Scheme 2).^69^ The reaction was postulated to proceed *via* spontaneous hydrolytic ring opening of the cyclic imines, thus liberating a carbonyl functionality that could act as an electron-withdrawing activating group for the C=C-double bond (enone-type). The reaction was coupled to an imine reductive step catalyzed by imine reductase (see Section 1.1.3). In the case where two chiral centers were generated consecutively, conversion to heterocyclic amines through a one-pot cascade set-up was high, however diastereoselectivities remain moderate.

**Scheme 7:**
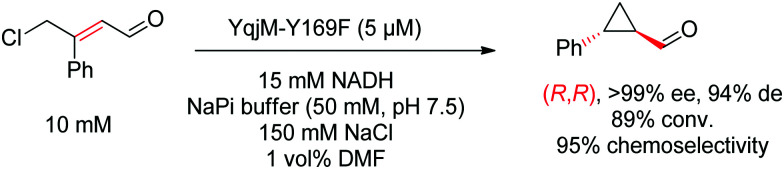
Reductive carbocyclization catalyzed by a tyrosine variant of YqjM.^70^

Mechanistically derived from the initial reductive step targeting the C=C-double bond of γ-halogenated α,β-unsaturated aldehydes and ketones, a reductive carbocyclization reaction could be selectively achieved using OYEs in which the catalytic proton donor had been silenced (mutation of tyrosine to phenylalanine).^70^ Advantage was taken of the intrinsic reactivity of the intermediate enolate generated, of the proximity of the electrophilic γ-center, and of the slow nonenzymatic protonation ‘quenching’ step, which all contributed to an intramolecular nucleophilic substitution leading to cyclization (Scheme 7). The reaction formed chiral 1,2-disubstituted cyclopropanes and diastereoselectivity was particularly high with the YqjM mutant Y169F.

**Scheme 8:**
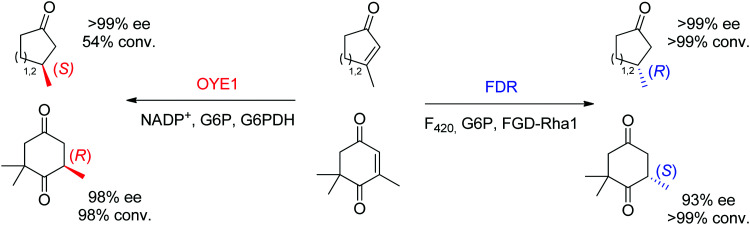
Stereocomplementary reduction of cyclic enones and ketoisophorone by OYE1^74^ and FDR^73^ (FDR: F_420_H_2_-dependent reductase, FGD-Rha1: F_420_-dependent glucose 6-phosphate dehydrogenase from *Rhodococcus jostii* RHA1, G6P: glucose 6-phosphate, G6PDH: glucose 6-phosphate dehydrogenase).

Finally, additional biocatalysts suitable for C=C-double bond reductions have been identified with nicotinamide-independent F_420_H_2_-dependent reductases (FDRs) from the superfamily of flavin/deazaflavin oxidoreductases (FDORs).^62,71^ While initially applied to the reduction of quinones, coumarins and pyrones to nonchiral compounds,^72^ reductases from *Mycobacterium hassiacum* and *Rhodococcus jostii* RHA1 lately showed excellent stereoselectivity in the reduction of β-substituted α,β-unsaturated aldehydes and cyclic enones.^73^ Despite overall low catalytic rates, these enzymes may turn relevant as stereocomplementary catalysts to OYEs in the reduction of particular substrates. For instance, ketoisophorone, invariably reduced to (*R*)-levodione by OYEs, was transformed into the (*S*)-enantiomer by FDRs. Importantly, the cofactor F_420_ can be recycled by a F_420_-dependent glucose 6-phosphate dehydrogenase at the expense of glucose 6-phosphate. As recently demonstrated, these enzymes also proceed *via* a *trans*-addition of [2H] and could reduce tetrasubstituted cyclic enones with high stereo- and diastereoselectivity.^61^ In selected cases, including the reduction of ketoisophorone and 3-methylcycloalk-2-enone, FDR appeared stereocomplementary to OYE1 (Scheme 8).^74^

#### 1.1.2. Carbonyl reduction

Alcohol dehydrogenases (ADHs) catalyze the stereoselective reduction of ketones at the expense of NAD(P)H and are found within diverse protein superfamilies:^75–78^ (i) the class of medium-chain dehydrogenases/reductases (MDR) typically relies on a zinc atom as a Lewis acid for activation of the carbonyl group. (ii) Short-chain dehydrogenases/reductases (SDR) do not require a metal for catalysis but in some cases (such as with ADHs from *Lactobacillus kefir* and *Lactobacillus brevis*), a magnesium may be necessary to retain activity, mostly due to structural reasons. These enzymes accept a broad range of substrates. (iii) Long-chain dehydrogenases/reductases (LDR) are diverse proteins that are not all metal-dependent enzymes, they act notably on polyol-type substrates. (iv) Aldo–keto reductases (AKR) finally are found in metabolic reactions of endogenous compounds and xenobiotics, and as a consequence, are known for their large substrate spectrum. Members of the MDR and the SDR superfamilies are broadly applied in stereoselective reductions of ketones toward enantiopure secondary alcohols and their successful implementation in industrial processes has been reported in several cases.^79–82^

While aldehydes are readily reduced by several ADHs, no chiral center is generated in the reaction and corresponding reactions will not be treated here. However, dynamic kinetic resolutions of racemic α-substituted aldehydes were successfully attained and are briefly discussed in Section 2.2. Since the reaction is reversible, ADHs are frequently used in oxidation reaction and can be implemented in enantioselective processes with chiral alcohols, such as in (dynamic) kinetic resolution, stereoinversion or cyclic deracemization.^83,84^

**Scheme 9:**
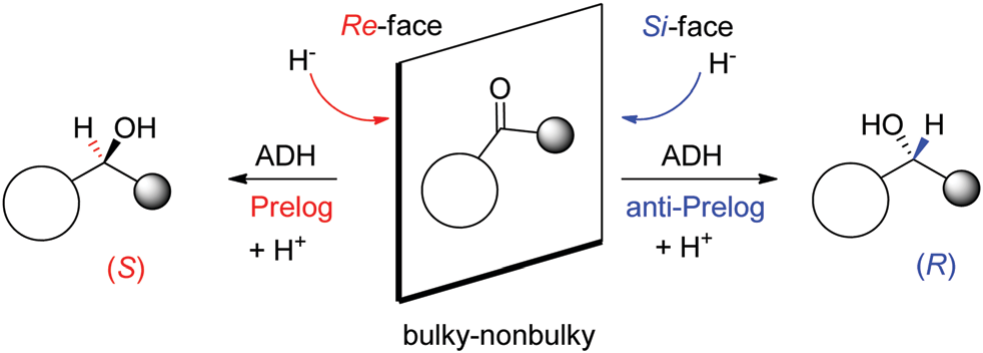
Prelog and anti-Prelog rule applied to the reduction of carbonyl compounds by alcohol dehydrogenases (ADH). H^−^ denotes the hydride of NAD(P)H, not shown for clarity. Republished from ref. 38 with permission of John Wiley & Sons – Books, copyright 2021; permission conveyed through Copyright Clearance Center, Inc.

A major reason for the broad acceptance of ADHs in the organic chemistry laboratory^85^ is the possibility to predict the stereochemical outcome of the reaction based on the choice of the enzyme and the configuration of the substrate (Scheme 9). So-called Prelog ADHs follow the Prelog rule,^86^ that is the carbonyl is attacked on the *Re*-face by the hydride of NAD(P)H and furnishes the (*S*)-alcohol, as long as the smallest substituent has the lowest priority according to the Cahn-Ingold-Prelog (CIP) sequence rule (used to name stereoisomers with fixed absolute configuration). Stereocomplementary enzymes denoted as anti-Prelog ADHs deliver, in the contrary and under same considerations, (*R*)-alcohols after attack on the *Si*-face.^87^

Owing to a fantastic protein diversity, ADHs display stereopreference on various substrate types. Not only bulky–nonbulky substrates with major size differences between the two substituents, but also bulky–bulky substrates such as diaryl ketones can be reduced to enantiopure alcohols.^80,88–92^

For particularly challenging substrates, such as large compounds or multifunctionalized molecules, the implementation of protein engineering methods can tailor a particular ADH for the targeted application.^93–96^ Such approach has been regularly employed under industrial setting and the resulting catalysts often displayed, in addition to perfect stereoselectivity, high catalytic activity as well as increased robustness toward process conditions.^97–99^

**Scheme 10:**
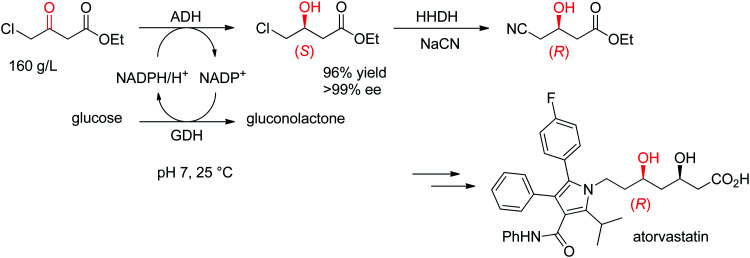
Enzymatic cascade for the asymmetric synthesis of the chiral precursor to atorvastatin based on an ADH-catalyzed step (GDH: glucose dehydrogenase, HHDH: halohydrin dehalogenase).^100^ Republished from ref. 38 with permission of John Wiley & Sons – Books, copyright 2021; permission conveyed through Copyright Clearance Center, Inc.

The statin side chain, with its two asymmetric hydroxylated centers, can be conveniently prepared by an ADH-catalyzed reductive strategy. In one synthetic approach to atorvastatin – a blockbuster drug used in the prevention of cardiovascular disease and the treatment of high levels of cholesterol – an enzymatic cascade was developed to access the key chiral precursor of the final active pharmaceutical ingredient (API). An ADH engineered by Codexis was applied to the reduction of 4-chloroacetoacetate into (*S*)-4-chloro-3-hydroxybutyrate, while the reduced nicotinamide cofactor was regenerated by glucose/glucose dehydrogenase (Scheme 10). The reaction was performed at pH 7 and 25 °C and yielded (*S*)-4-chloro-3-hydroxybutyrate in 96% yield and >99.5% ee. This intermediate was then further converted in a sequence of enzymatic dehalogenation–epoxidation catalyzed by a halohydrin dehalogenase and chemical cyanolysis to deliver (*R*)-4-cyano-3-hydroxybutyrate, which could be incorporated into the final API.^100,101^

**Scheme 11:**
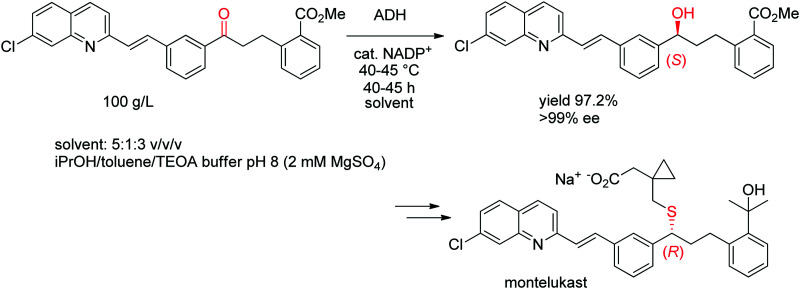
Asymmetric synthesis of the (*S*)-precursor to montelukast by ADH-catalyzed reduction (ADH: engineered alcohol dehydrogenase, TEOA: triethanolamine).^102^ Republished from ref. 38 with permission of John Wiley & Sons – Books, copyright 2021; permission conveyed through Copyright Clearance Center, Inc.

A chemoenzymatic process has been developed by Merck & Co, Inc. for the synthesis of montelukast, an anti-asthmatic agent. The key step of asymmetric biocatalytic reduction replaced the chemical synthesis that was based on a chiral reducing agent and could provide the crucial (*S*)-alcohol intermediate en route to montelukast in enantiopure form. An ADH was engineered by Codexis in several rounds of directed evolution to accept the bulky poly-functionalized substrate [(*E*)-methyl 2-(3-(3-(2-(7-chloroquinolin-2-yl)vinyl)phenyl)-3-oxopropyl)benzoate] under challenging reaction conditions (Scheme 11). The engineered biocatalyst was finally employed in 67 vol% organic solvent in form of a mixture of isopropanol and toluene over 40–45 h reaction time at up to 45 °C on 230 kg scale at 100 g L^−1^ substrate, whereby isopropanol served as hydride source *via* the coupled-substrate cofactor recycling approach.^102^

Recently, promiscuous reductive activity was found in the Baeyer–Villiger monooxygenase cyclohexanone monooxygenase (CHMO) from *Acinetobacter* sp. NCIM 9871. Initially, the wild-type enzyme showed only poor activity in the reduction of an aromatic α-keto ester substrate under anaerobic conditions. Rational design based on site-directed mutagenesis was therefore applied and led to the generation of a highly efficient and selective biocatalyst that followed the anti-Prelog rule on a range of aromatic α-keto esters. Importantly, in the case of this artificial ketoreductase, the hydride delivery was suggested to proceed through the reduced FAD, akin to the mechanism of Old Yellow Enzymes in C=C-double bond reduction reactions (see Section 1.1.1).^103^

#### 1.1.3. Imine reduction/reductive amination

**Scheme 12:**
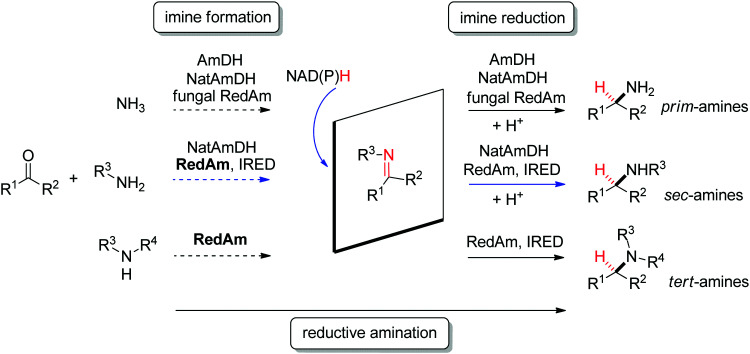
Stereoselective enzymatic strategies in imine reduction to access primary, secondary and tertiary chiral amines. An enzyme-catalyzed imine formation may be included, rendering a reductive amination. Hydride attack of the imine intermediate depicted arbitrarily for the case of primary amine (blue arrow) as amine substrate. Dashed arrows indicate that the reaction can be enzymatically catalyzed (IRED: imine reductase, (Nat)AmDH: (native) amine dehydrogenase, RedAm: reductive aminase).

**Fig. 2:**
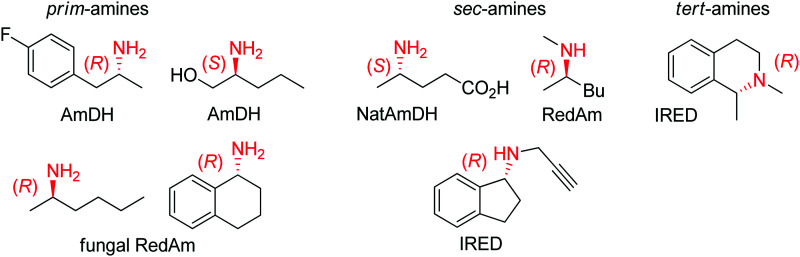
Sampling of products obtained by biocatalytic reductive amination, except tertiary amines, which were obtained by enzymatic iminium reduction only (IRED: imine reductase, (Nat)AmDH: (native) amine dehydrogenase, RedAm: reductive aminase).^114,115,119–123^

Several types of enzymes have been identified for the stereoselective reduction of imines (Scheme 12 and Fig. 2).^104^ The most established biocatalysts responsible for C=N reduction in presence of NADPH are imine reductases.^105,106^ The field of enzymatic imine reduction is a prime example of how rapidly the field of biocatalysis can evolve.^107^ Within the last decade, the portfolio of imine reductases (often denoted IREDs) grew from a few examples known from specific biosynthetic pathways with little application potential in biocatalysis – most prominent being the case of dihydrofolate reductase involved in the synthesis of tetrahydrofolic acid – to large collections of stereocomplementary enzymes, for which specific sequence motifs could be identified.^108^ The stereoselectivity has been assigned to two distinct stereocomplementary superfamilies.^109^ New imine-reducing enzymes can also be obtained by introducing mutations in closely related enzyme classes, as was shown with β-hydroxyacid dehydrogenases. An engineered enzyme bearing two mutations was found highly stereoselective in the reduction of 6-phenyl-2,3,4,5-tetrahydropyridine, yielding the corresponding (*S*)-product with 97% ee, thereby outperforming wild-type IREDs.^110^

Imine reductases have been preferentially applied to the reduction of cyclic imines,^111–113^ which present a higher stability in aqueous reaction media. By including *in situ* formation of the imine substrates in the reaction scheme, exocyclic and acyclic imines can also be converted to chiral secondary amines. To favor the reversible formation of the imine in aqueous media, a large excess of the amine in comparison to the carbonyl compound is employed. Since both imine and iminium ion are accepted, IREDs can be employed for the formation of secondary and tertiary amines (Fig. 2).^114–116^

In addition, the C=N reduction reaction may be enzymatically coupled to preceding catalyzed imine formation. The resulting enzymatic reductive amination of carbonyl compounds with primary amines could be observed with some imine reductases.^117,118^ However, to reach synthetically relevant conversion levels, a large excess of the amine substrate is necessary, poising both the atom economy of the reaction and downstream processing. Since chemoselectivity is high and the carbonyl substrate cannot be depleted by alternative reductive pathways, this approach is still very attractive and advantageous compared to some general chemical approaches.

A subclass of imine reductases called reductive aminases (RedAms) was later shown capable of catalyzing the imine formation more efficiently, rendering a biocatalytic reductive amination of carbonyl compounds with primary and secondary amines.^122,124^ This system does not require an excess of amine and offers a more attractive synthesis of secondary and tertiary amines enabled by the use of near-stoichiometric carbonyl/amine equivalents. Although ammonia was not well accepted by the first identified reductive aminases, the discovery of fungal RedAms accepting the simplest amine donor considerably enlarged the possibility to obtain primary amines by enzymatic reductive amination, a synthetic advantage that was further strengthened by increased thermostability of the proteins.^123^ Recently, a large set of imine reductases were shown to operate at a close to 1 : 1 stoichiometry (amine/carbonyl), with only a few enzymes being categorized as reductive aminases. This highlights the difficulty in assigning activity in reductive amination at low stoichiometric excess to particular imine reductases according to their structure and sequence similarities. In this work, the authors importantly identified anilines and other heteroaromatic amines as amine partners.^125^

Overall, sequence-activity relationships with imine reductases are highly substrate-dependent and no general rule could be delineated to anticipate the outcome of a particular reaction or from specific pairs of substrate/enzyme.^126^

IREDs can be further creatively combined with chemical steps, as shown by combining an enzymatic imine reduction with a base-mediated rearrangement to generate chiral 2,2-disubstituted azepanes and benzazepines.^127^

**Scheme 13:**
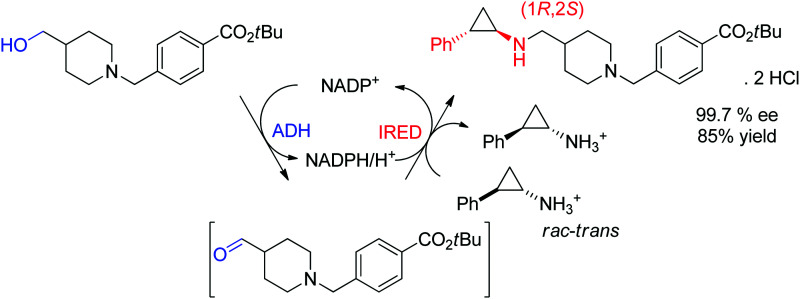
Application of imine reductase (IRED) in the kinetic resolution of a racemic chiral amine substrate *via* reductive amination in an enzymatic cascade for formal amination of a primary alcohol.^128^

An example of application of IREDs in industry was reported by GSK for the synthesis of a molecule currently in clinical trials for use in the treatment of leukemia. Here, the chiral information was already present in the amine substrate, which was used as racemic starting material and coupled to an aldehyde by enzymatic reductive amination (Scheme 13). After three rounds of directed evolution, excellent catalytic and process performance was obtained for the selected imine reductase. This kinetic resolution was performed with the engineered enzyme on 20 L scale at acidic pH (4.6) and delivered the chiral secondary amine product with 99.7% ee in almost 85% isolated yield.^128^ Importantly, the green metrics of the process could be considerably improved compared to previous chemical approach. Finally, the reaction was elegantly installed in a redox-neutral cascade,^129^ in which the aldehyde was obtained by oxidation of the corresponding alcohol precursor by an alcohol dehydrogenase, thus rendering a formal amination of primary alcohol^130,131^ in presence of catalytic amounts of the nicotinamide cofactor and at equimolar amine loading.

Other success stories have contributed to extend the list of biocatalysts used in the reductive amination of highly diverse ketone substrates. The first breakthrough came from the engineering of amino acid dehydrogenases, which naturally catalyze the reversible reductive amination of α-ketoacids with ammonia and have a long history in the asymmetric synthesis of amino acids.^132^ During a protein engineering campaign, and by targeting few crucial residues responsible especially for binding of the amino acid substrate, Bommarius and coworkers could abolish the requirement of leucine dehydrogenase for the substrate carboxylic acid moiety used as anchor. This provided the first example of engineered amine dehydrogenases (AmDHs) accepting ‘simple’ ketones for the synthesis of primary amines.^133,134^ Soon after, other leucine dehydrogenases and phenylalanine dehydrogenases were mutated in a comparable manner and provided a range of efficient biocatalysts to access chiral primary amines in enantiopure form.^135^

Conceptually related was the engineering of an ε-deaminating l-lysine dehydrogenase into an AmDH. Through introduction of only one mutation, the requirement for the terminal α-amino acid functionality, although not so strict with the native enzyme, was lost and the new biocatalyst could efficiently accept a range of ketones in asymmetric reductive amination reaction with ammonia, including aromatic substrates at up to 100 mM concentration and 50 °C. Rather astonishing here was that the native enzyme accepts an aldehyde as natural carbonyl substrate and therefore did not 'need' to display stereoselectivity in the first place. The enzyme was successfully applied to the reductive amination of acetophenone on 600 mg scale using ammonium formate, and the final (*R*)-amine could be obtained in perfect enantiopurity and 85% isolated yield. A formate dehydrogenase was employed to regenerate the nicotinamide cofactor.^136^

The scope of biocatalytic reductive amination was finally recently enlarged through the discovery of a family of stereoselective native amine dehydrogenases for the synthesis of primary and secondary amines. As in the case of engineered AmDHs, a large excess of ammonia is employed, typically in form of ammonium formate, while the necessary nicotinamide cofactor is used in catalytic amounts and recycled *in situ* by a regeneration system such as glucose/glucose dehydrogenase.^121,137^

As opposed to imine reductases and reductive aminases, engineered AmDHs are specific for NADH, while native AmDHs show slight preference for NADPH.

Finally, the case of opine dehydrogenase deserves mention. The native enzyme accepts α-keto acids and couples them with α-amino acids.^138^ Codexis evolved this enzyme toward an active biocatalyst for the reductive amination of ketones with a range of secondary amines. In many cases, chiral tertiary amines could be obtained with excellent stereoselectivity.^139^

### 1.2. C–Heteroatom-bond formation

#### 1.2.1. C–N-Bond formation

##### 1.2.1.1. Transamination of C=O-bond

**Scheme 14:**
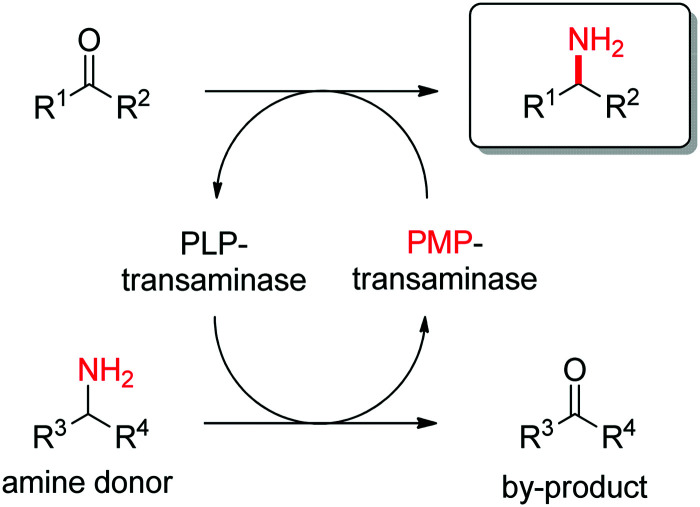
Overall concept of amino-transfer using PLP-dependent transaminase (PLP: pyridoxal-5′-phosphate, PMP: pyridoxamine-5′-phosphate).

Transaminases catalyze the formal swap of functional groups *via* amino-transfer between a carbonyl acceptor and an amino donor, which means that for amine synthesis, a ketone is formed as by-product. These aminotransferases are pyridoxal-5′-phosphate (PLP)-dependent enzymes and use the cofactor as amine shuttle between the donor and the acceptor. In the amination direction, PLP first gets aminated by the amine donor to form pyridoxamine-5′-phosphate (PMP), which then transfers the amino group to the carbonyl substrate (Scheme 14).^140^ For applications in biocatalysis, so-called ω-transaminases have been lately dominating (later shortly denoted transaminases). These enzymes accept aliphatic ketones as substrates, without requirement for other functional groups.^141–143^

**Scheme 15:**
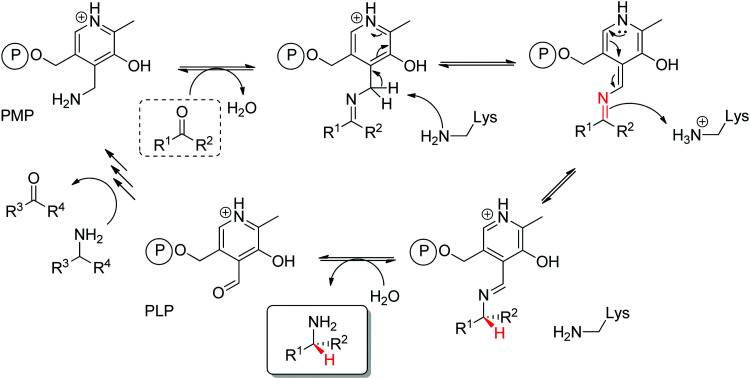
Mechanism of transaminase for the amination of a carbonyl compound (PLP: pyridoxal-5′-phosphate, PMP: pyridoxamine-5′-phosphate). The (first) steps of amination of PLP to PMP are not detailed for clarity (*e.g.*, PLP bound to the enzyme as an internal aldimine *via* a lysine residue before formation of the external aldimine with the amine donor en route to PMP formation).

Prochiral ketones are converted stereoselectively to primary amines. Mechanistically, the face recognition occurs at the stage of the planar quinonoid ‘imine-type’ intermediate generated between the substrate carbonyl and the aminated cofactor (PMP), which locks the substrate in a defined position; the change from prochiral to chiral molecule occurs through stereoselective protonation of this intermediate by a conserved lysine (Scheme 15). Both (*R*)- and (*S*)-selective transaminases have been identified^144^ and while the protein folds differ, the stereocomplementarity can be simply explained by the different positioning of the lysine in relation to the cofactor binding pocket: the key catalytic residue is situated on the *Si*-face of the PLP in (*S*)-transaminases and on the *Re*-face in (*R*)-transaminases.^145–148^ Determinant for the stereo-outcome of the reaction is thus the reaction of the carbonyl with the aminated cofactor and generation of the prochiral imine intermediate.

While considered only a decade ago ideal biocatalysts for the preparation of chiral amines, the field has developed in such an incredible manner over the last few years that other options that do not suffer from equilibrium limitations typical for transfer reactions and from low atom economy due to inherent generation of by-products are rapidly becoming prevalent in the laboratory (*e.g.*, amine dehydrogenases and reductive aminases, see Section 1.1.3). Importantly, major efforts in the field have delivered suitable solutions to render transaminase-catalyzed reactions attractive in synthesis.^149^ To favor the amination reaction, strategies involve for instance the (enzymatic) removal of the ketone co-product, the use of 'smart' amine donors (*e.g.*, 1,2-diamines that dimerize after one amine transfer reaction^150^), or the regioselective conversion of diketones to yield imines followed by reduction to the corresponding cyclic amines.^151,152^

**Scheme 16:**
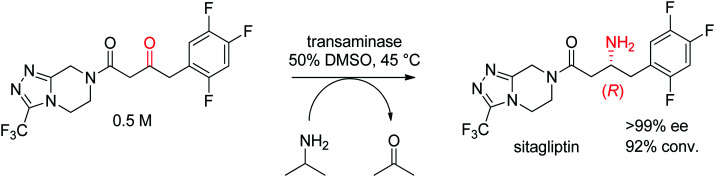
Transaminase-catalyzed amine transfer developed by Merck & Co, Inc. to access (*R*)-sitagliptin in optically pure form.^153^

The industrial biocatalytic synthesis of the antidiabetic drug sitagliptin by Merck & Co, Inc. is a testimony of the strong synthetic value of transaminases for accessing primary chiral amines. The ketone precursor is a bulky multifunctionalized molecule. Protein engineering was necessary to reach both high level of activity and stereoselectivity and render the transaminase amenable to application under process conditions (Scheme 16). The engineered biocatalyst displayed 27 mutations and could convert the ketone at high substrate concentration (0.5 M and 1 M of isopropylamine as amino donor) in 50% DMSO at 45 °C with perfect stereoselectivity (>99% ee), furnishing the final product in high yield (>90%). This example quickly became a benchmark for industrial biotransformations.^153,154^

##### 1.2.1.2. Hydroamination of C=C-bond

Enzymatic hydroaminations of alkenes are performed by ammonia lyases. The enzymes catalyze the reversible addition of ammonia across C=C-double bonds of α,β-unsaturated carboxylic acids. The reaction is stereoselective and leads to the formation of chiral α-amino acids, however, to favor the synthetic direction, large excess of ammonia is necessary.^155^ The case of amino mutases will not be discussed here. These mechanistically diverse isomerases transfer the amino group of α-amino acids to the β-position *via* a formal 1,2-shift, yielding chiral β-amino acids; while the reaction is stereoselective, the chiral information is not necessarily retained.^156^

Prevalent ammonia lyases include aromatic ammonia lyases phenylalanine and tyrosine ammonia lyases (PAL and TAL), and (methyl)aspartate ammonia lyases. Several of these enzymes can accept a range of unsaturated (di)carboxylic acids beyond their natural substrate.^157,158^

**Scheme 17:**
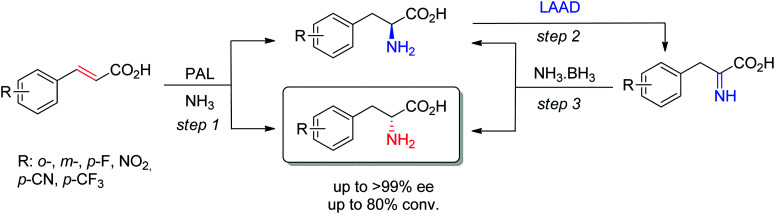
Access to d-arylalanines in a cascade through enzymatic nonselective hydroamination and cyclic deracemization combining enantioselective oxidation and chemical reduction (LAAD: l-selective amino acid deaminase, PAL: phenylalanine ammonia lyase).^160^

PAL has been employed for instance for the preparation of a range of (nonnatural) aromatic amino acids starting from 3-arylacrylates with various substitution. For instance, several (*S*)-halophenylalanines could be obtained with excellent enantiopurity and high space time yields (up to >200 g L^−1^ d^−1^) by employing a cyanobacterial PAL with ammonium carbamate (4 M) as reaction buffer at high substrate loadings (>0.3 M).^159^

d-Arylalanines cannot be obtained with high stereoselectivity by PAL. The nonperfect stereoselectivity of a newly identified wild-type enzyme could be exploited to access d-arylalanines with high enantiopurity. The concept (Scheme 17) relied on the low selectivity in the hydroamination reaction (step 1), combined with a kinetic resolution of the nonenantiopure amino acids through enantioselective oxidation to the corresponding imino acid by an l-amino acid deaminase (step 2), followed by nonselective chemical reduction back to the amino acid (step 3). Steps 2–3 allowed enrichment of the d-amino acid products through a cyclic deracemization, finally furnishing high ee values after multiple cycles. Reactions performed on 100 mL allowed access to 50–60 mg of isolated products.^160^

In selected cases, primary amines are also accepted in place of ammonia, as was shown in the synthesis of *N*-substituted l-aspartic acids using for instance mutated methylaspartate ammonia lyase on fumarate.^161–163^

**Scheme 18:**
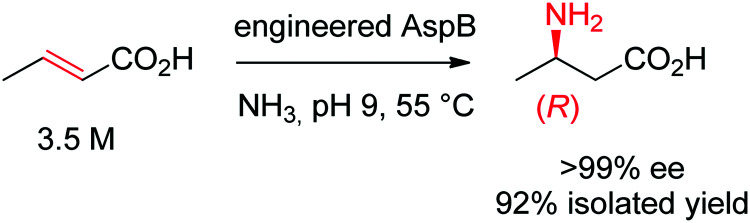
Hydroamination of crotonic acid to (*R*)-β-aminobutanoic acid by an engineered aspartate ammonia lyase AspB from *Bacillus* sp. YM55-1.^165^

Asymmetric direct β-hydroamination of α,β-unsaturated carboxylic acids by ammonia lyases was made possible through computational redesign of aspartate ammonia lyase AspB from *Bacillus* sp. YM55-1. The natural enzyme was highly specific for the dicarboxylic acid substrate fumarate, respectively for aspartate in the deamination direction. By employing the algorithm Rosetta Enzyme Design initially developed for *de novo* protein design,^164^ the requirement for a second carboxylic acid moiety could be abolished. The best resulting variants successfully transformed a range of substituted acrylates into the corresponding aliphatic and aromatic β-amino acids with high stereoselectivity. The reaction was run on a kg-scale at 300 g L^−1^ concentration of crotonic acid at pH 9 and 55 °C and selectively yielded (*R*)-β-aminobutanoic acid with 99% conversion and >99% ee (Scheme 18).^165^

By combining several enzymes in a one-pot cascade, a formal hydroamination of nonactivated alkenes was developed. The sequence epoxidation–isomerization–transamination was achieved with α-methyl styrene by the action of styrene monooxygenase, styrene oxide isomerase and transaminase. The three enzymes, plus alanine dehydrogenase used to drive the amination reaction, were co-expressed heterologously in *E. coli*, and the resulting biocatalyst employed as whole cells could catalyze the formal anti-Markovnikov selective formation of (*S*)-2-phenylpropan-1-amine in up to 97% ee.^166^ This elegant approach demonstrates the maturity of the field, since formal reactions can now be achieved in one pot by smartly combining individual enzymes in cascade reactions.

#### 1.2.2. C–O-Bond formation

##### 1.2.2.1. Alkene epoxidation

**Scheme 19:**
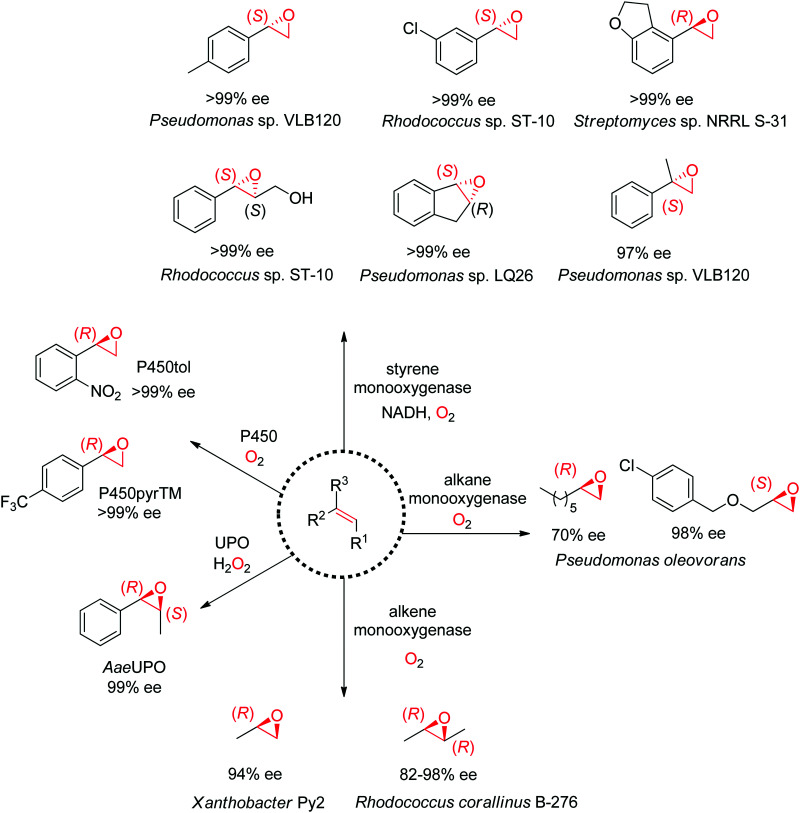
Enzymatic platform for the stereoselective epoxidation of alkenes and examples of products formed (UPO: unspecific peroxygenase). P450pyrTM: triple mutant of P450 monooxygenase from *Sphingomonas* sp. HXN-200, P450tol: P450 monooxygenase from *Rhodococcus coprophilus* TC-2, *Aae*UPO: UPO from *Agrocybe aegerita*.

A range of enzymes are available to catalyze the asymmetric *cis*-epoxidation of C=C-double bonds *via* formal insertion of oxygen (Scheme 19). The reaction is particularly valuable as the oxirane ring may be opened through various (nucleophilic) substitution reactions, including enzymatic routes, thereby offering access to hydroxy-functionalized compounds with up to two chiral centers. The stereoselective attack on one face of the C=C-bond requires oxygen activation, and this is achieved by diverse cofactor-dependent enzymes, such as flavin-dependent monooxygenases, iron-dependent nonheme monooxygenases and iron-heme dependent monooxygenases (P450 enzymes).^167^ Exception are heme-thiolate (fungal) peroxygenases, which use hydrogen peroxide directly as oxidant.^168^ The substrate spectrum depends heavily on the type of biocatalysts.

**Scheme 20:**
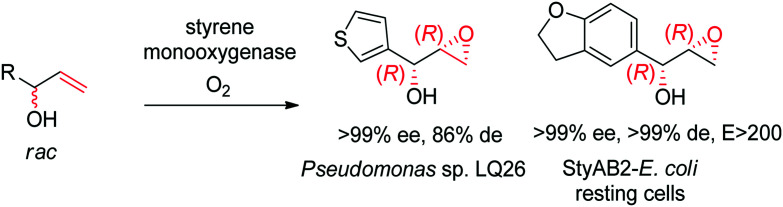
Kinetic resolution of racemic allylic alcohols by styrene monooxygenase-catalyzed epoxidation.^173^

Despite being some of the most widely applied systems in enzymatic epoxidation reactions, styrene monooxygenases are not simple biocatalysts. Considered a prototype enzyme of this class of flavoenzymes, styrene monooxygenase from *Pseudomonas* sp. is composed of two components: the NADH-dependent flavin reductase StyB catalyzes the necessary activation of the flavin of the FAD-dependent epoxidase StyA through reduction with nicotinamide. The reduced FAD then transfers to StyA, where it reacts with molecular oxygen to generate a C(4a)-peroxyflavin intermediate, which can attack the alkene substrate, usually in a highly stereoselective manner.^169^ To simplify the reaction set-up, whole cells of *E. coli* recombinantly expressing the two components are employed and provide sufficient amount of NADH for the initial reductive step, while air serves as the source of oxidant. Poorly functionalized small molecules are best accepted, a clear advantage compared to traditional chemical methods, and typically yield (*S*)-epoxides. A range of α- and β-substituted styrenes, as well as terminal and cyclic alkenes, have been successfully epoxidized with the StyA/StyB system. In addition to substituted styrenes, 1-phenylethenes are good substrates for styrene monooxygenases (Scheme 19).^170–172^ Exquisite enantioselectivity has been observed on racemic substituted allylic alcohols, which, combined to the perfect stereoselectivity of the epoxidation, led to a kinetic resolution toward the formation of oxirane-containing secondary alcohols with excellent de values. *E* values were in several cases >200 (Scheme 20).^173^

Biocatalytic cascades initiated by styrene monooxygenase-catalyzed epoxidation reaction have allowed the functionalization of styrenes beyond epoxidation and generated in one pot chiral α-hydroxy acids and α- and β-amino acids.^174,175^

An exception to the (*S*)-selective styrene monooxygenases was recently identified with the characterization of an (*R*)-selective styrene monooxygenase from *Streptomyces* sp. Only the oxygenase component A could be identified through genome mining, and when coupled to the reductase component B from *Pseudomonas* sp., it showed activity on a few styrene derivatives, which were all converted to the corresponding (*R*)-epoxides with ee values up to >99%.^176^ The primary sequence of this protein was then used to perform searches in protein databases and led to the identification of eight (*R*)-selective styrene monooxygenases with enlarged substrate scope.^177^ Notably, from a phylogeny perspective, the (*R*)-selective enzymes appear grouped in a clade away from the (*S*)-selective ones.

Nonheme monooxygenases that depend on iron include membrane-bound xylene monooxygenases^178,179^ – with activity pattern comparable but not superior to that of styrene monooxygenases – and alkane and alkene monooxygenases (Scheme 19). Alkane monooxygenase from *Pseudomonas oleovorans*, which also catalyzes the hydroxylation of alkanes, contains three components – the nonheme iron ω-hydroxylase and the two-component electron-transport chain system – and is thus practically employed as whole-cell biocatalyst. The enzyme works well with linear terminal alkenes. (*R*)-Epoxides are usually obtained.^180–182^ Alkene monooxygenases are also multi-component biocatalysts and preferentially take short-chain alkenes as substrates.^183,184^

Catalytically versatile P450 monooxygenases rely on an iron(iv)-oxo porphyrin-cation-radical species (so-called ‘compound I’) for the oxygen atom transfer. The substrate spectrum is broad and includes diverse molecules such as terminal alkenes, unsaturated fatty acids and styrene derivatives. The major limitation is common to catalysis by P450s: the low substrate concentration tolerance limits their applications in preparative synthesis. Importantly, P450s are usually stereocomplementary to styrene monooxygenases and (*R*)-styrene epoxides are obtained. Several P450-based systems have been disclosed. For instance, natural P450tol showed exquisite stereoselectivity on a variety of styrenes,^185^ while P450pyr could be specifically engineered to deliver (*R*)-styrene epoxides with excellent enantiopurity (Scheme 19).^186^ The engineered triple mutant could also effectively convert *ortho*-substituted styrenes with (*S*)-selectivity, 1,1-disubstituted alkenes as well as cyclic alkenes. Lately, an engineered P450BM3 employed in the presence of a dual functional small molecule could produce (*R*)-styrene epoxide in the peroxygenase mode.^187^

Systems based on heme-dependent unspecific peroxygenases (UPOs) that utilize hydrogen peroxide as oxidant are currently emerging.^188,189^ A few of these enzymes mostly originating from fungi have been well characterized and include UPOs from *Agrocybe aegerita* (*Aae*UPO),^190^*Coprinellus radians* (*Cra*UPO)^191^ and *Marasmius rotula* (*Mro*UPO).^192^ A major attractive feature of these biocatalysts is their catalytic efficiency, translating into high TTN values (up to 110 000), coupled with exquisite stereoselectivity, as seen in the *cis*-epoxidation of styrene derivatives (Scheme 19).^193^ As with P450 enzymes, the tolerance to high substrate concentration remains poor, which currently prevents their use in large scale applications. Finally, the heme center is poorly compatible with the oxidant; this problem however can be partly circumvented by *in situ* H_2_O_2_ production strategy.^194^

**Scheme 21:**
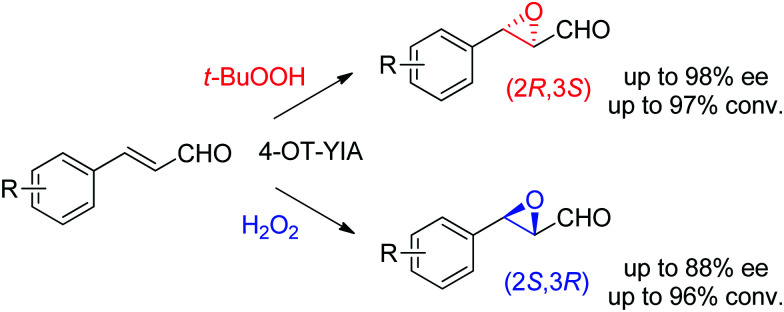
Stereocomplementary epoxidation of α,β-unsaturated aldehydes *via* switch of oxidant with a triple variant of 4-oxalocrotonate tautomerase (4-OT).^196^

Lastly, the engineering of 4-oxalocrotonate tautomerase (4-OT) delivered remarkable catalytic promiscuity. The designed protein could accept different (organic) hydroperoxides to catalyze the epoxidation of a range of α,β-unsaturated aldehydes. As in the case of the Michael addition of nitromethane on α,β-unsaturated aldehydes catalyzed by 4-OT^195^ (see Section 1.3.2.1), the epoxidation reaction was suggested to proceed *via* an enzyme-bound iminium intermediate. Stereocomplementary versions using 4-OT displaying three mutations were obtained by changing the oxidant from *t*-BuOOH for attack on the *Re*-face, to hydrogen peroxide for attack on the *Si*-face of the alkene, and granted access to epoxides with ee values up to 98%; diastereoselectivity was in most cases good to very good (Scheme 21).^196^ In both cases, the reaction was scalable and was combined with a chemical reduction by NaBH_4_ to deliver the corresponding epoxy-alcohols.

##### 1.2.2.2. Alkene hydration

A few enzymes can be employed to catalyze the asymmetric hydration of alkenes, a challenging reaction for chemists. Unactivated alkenes can be converted to the corresponding alcohols by linalool dehydratase isomerase, carotenoid-1,2-hydratases or fatty acid hydratases, however, many of these hydratases are mostly active on a small subset of (natural) substrates.^197^ The oleate hydratase from *Elizabethkingia meningoseptica* was engineered to catalyze the stereoselective hydration of terminal and internal alkenes devoid of a carboxylic acid group. In the presence of a carboxylic acid decoy molecule, a range of secondary (*S*)-alcohols were obtained with excellent stereoselectivity (up to 99% ee).^198^

Cofactor independent ferulic acid decarboxylase (FDC) is broadly defined as a phenolic acid decarboxylase. The enzyme catalyzes the reverse decarboxylation of phenolic acids, a feature which has been exploited in biocatalysis for carboxylation reactions of nonnatural substrates.^199^ Catalytic promiscuity of FDC from *Enterobacter* sp. could be identified in the asymmetric hydration of hydroxystyrene derivatives.^200,201^ A range of (*S*)-4-(1-hydroxyethyl)phenols were obtained with ee values up to 71% and conversion up to 82%.

### 1.3. C–C-Bond formation

Several enzymatic strategies are available for asymmetric C–C-bond forming reactions^202,203^ and involve attack of a reactive (nucleophilic or radical) intermediate species onto a sp^2^ hybridized carbon atom, either within a C=O- or C=C-double bond (Scheme 1). The nucleophiles are diverse and include enolate/enamine, umpoled carbonyl carbon, cyanide and nitroalkane. Examples with more specialized enzymes have been reviewed.^204,205^

#### 1.3.1. Attack of C=O-bond

**Scheme 22:**
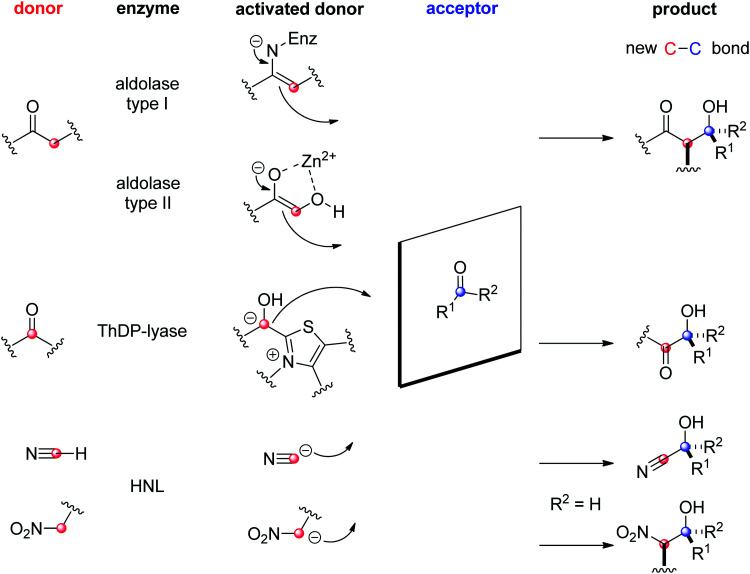
Enzymatic strategies for stereoselective C–C-bond formation with a carbonyl acceptor (HNL: hydroxynitrile lyase, ThDP: thiamine diphosphate).

The field of biocatalytic C–C-bond forming reactions using carbonyl as electrophile has long been dominated by lyases and aldolases for the synthesis of α- and β-hydroxy carbonyl derivatives, respectively (Scheme 22).^206,207^ Although both enzymes couple two carbonyl compounds together, they use very distinct catalytic machinery to that end, which explains the different nature of the products formed. Lyases rely on the thiamine diphosphate (ThDP) cofactor to perform an astonishing Umpolung reaction with the donor substrate, usually an aldehyde, but certain enzymes employ α-ketoacids such as pyruvate in decarboxylative carboligation reactions. The now nucleophilic carbonyl atom attacks the acceptor, (almost) invariably an aldehyde, yielding α-hydroxy ketones (acyloins). Aldolases, on the other hand, can activate the α-carbon of ketone donors *via* two distinct mechanisms:^208,209^ type I aldolases make use of a catalytic lysine to generate a reactive enamine intermediate, while type II aldolases require Zn^2+^ as a Lewis acid to activate the (often α-hydroxy functionalized) ketone donor *via* an enolate. The electrophilic acceptor was until recently always an aldehyde (*vide infra*).

The asymmetric synthesis of tertiary alcohols through C–C-bond forming reactions appeared possible with the identification of a ThDP-dependent enzyme that could accept ketones as acceptors. YerE from *Yersinia pseudotuberculosis* showed high stereoselectivity in the reaction of pyruvate with cyclic and open-chain ketones as well as ketoesters.^210^ More recently, highly selective access to tertiary alcohols could be granted by aldolases. Rhamnulose-1-phosphate aldolase (RhuA) from *Bacteroides thetaiotaomicron* employed dihydroxyacetone phosphate as nucleophile. The reactions with hydroxy functionalized ketones proceeded with high stereo- and diastereoselectivity to form the final products with two chiral centers.^211^ Later, and following previous indications that such reaction was indeed possible,^212,213^ a pyruvate aldolase was confirmed to accept ketones as electrophile with high stereoselectivity.^214^

Recently, biocatalytic formation of quaternary stereocenters by aldol addition of 3,3-disubstituted 2-oxoacid derivatives to aldehydes was demonstrated using metal-dependent 3-methyl-2-oxobutanoate hydroxymethyltransferase (KPHMT) that functioned as a type II aldolase. With some chiral nucleophiles, the reaction was enantioselective. The authors suggested that the formation of the (*E*)-enolate intermediate reactive species was favored, while the attack of the aldehyde was stereoselective, eventually yielding stereopure quaternary carbons. In the case of aldehydes other than formaldehyde, an additional chiral center was formed.^215^

Hydroxynitrile lyases (HNLs) have been investigated for a long time as catalysts in cyanohydrin formation reactions. An aldehyde or a ketone is being stereoselectively attacked by cyanide as the nucleophile and forms chiral α-hydroxynitriles. Stereocomplementary enzymes exist: (*R*)-selective HNLs are predominantly found in cyanogenic plants, including several *Prunus* species (such as those popularly known as apricot, plum, cherry and almond),^216,217^ or (noncyanogenic) weed plant *Arabidopsis thaliana*.^218^ One of the most investigated (*S*)-selective counterparts was identified in the rubber tree (*Hevea brasiliensis*).^219^ The substrate scope is broad and highly dependent on the enzyme.^220^

**Scheme 23:**
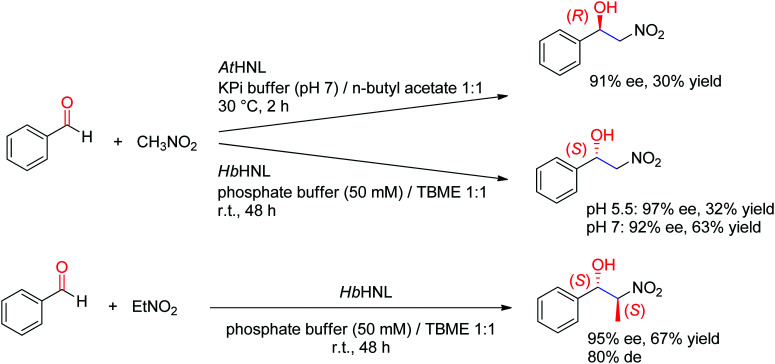
Biocatalytic asymmetric Henry reaction employing hydroxynitrile lyases (HNL). *At*HNL: HNL from *Arabidopsis thaliana*,^223^*Hb*HNL: HNL from *Hevea brasiliensis* (TBME: *t*-butyl methyl ether).^221^

A major addition to the reaction portfolio of HNLs was the development of a biological Henry reaction, in which nitroalkanes react as nonnatural nucleophiles with aldehydes to generate (*S*)-β-nitro alcohols (Scheme 23). The first example was demonstrated with nitromethane and high ee values for the products were obtained (92% ee in the case of benzaldehyde). Higher ee values could be obtained at acidic pH values (pH 5.5). With nitroethane, two chiral centers are generated, however despite high stereoselectivity in the nucleophilic attack of the C=O-double bond (95% ee), the *anti*-diastereoselectivity on benzaldehyde was only moderate (80% de).^221,222^ An (*R*)-stereocomplementary homologue was identified with HNL from *Arabidopsis thaliana*, which accepted nitromethane as donor and a range of substituted aldehydes as acceptors.^223^

#### 1.3.2. Attack of C=C-bond

##### 1.3.2.1. Activated alkenes (Michael addition)

**Scheme 24:**
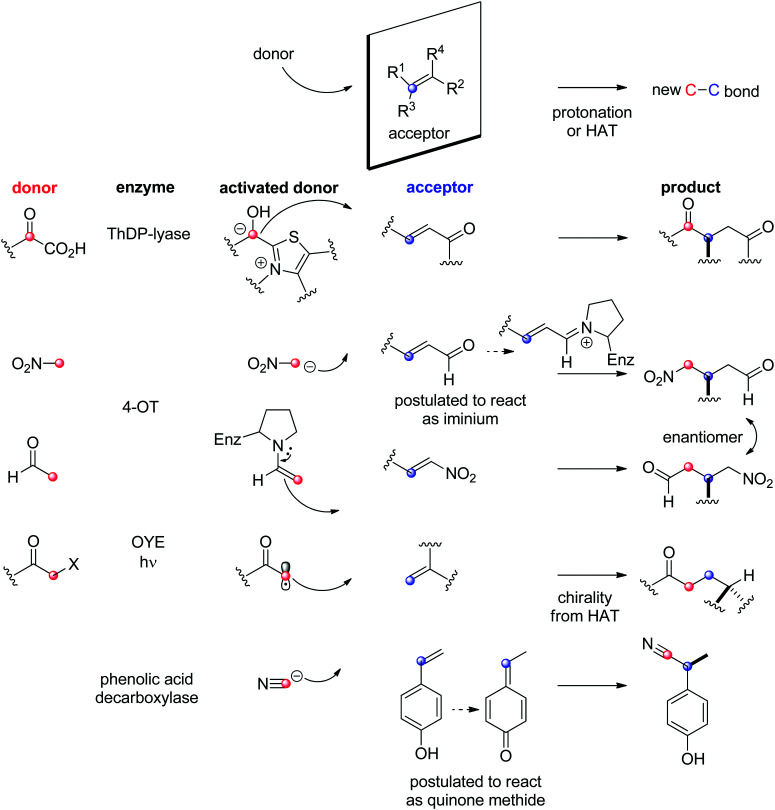
Enzymatic strategies for stereoselective C–C-bond formation with an alkene acceptor (4-OT: 4-oxalocrotonate tautomerase, HAT: hydrogen atom transfer, OYE: Old Yellow Enzyme, ThDP: thiamine diphosphate).

Enzymatic asymmetric versions of the so-called Michael addition – the nucleophilic attack of a C=C-double bond within an activated alkene – include different scenarios, depending on the nature of both the nucleophilic donor and the electrophilic acceptor (Scheme 24).

With ThDP-dependent lyases, an exception in the type of acceptor generally accepted (see Section 1.3.1) was discovered, and examples of a biological Stetter reaction were identified with the decarboxylative Michael addition of pyruvate onto α,β-unsaturated ketones. PigD from *Serratia marcescens* for instance catalyzed the 1,4-addition of pyruvate on a range of aliphatic and (hetero)aromatic α,β-unsaturated ketones with perfect chemo- and stereoselectivity to produce 2-substituted 1,4-diketones. Besides pyruvate, also 2-oxobutanoate was accepted as donor.^224,225^ An intramolecular nondecarboxylative version was developed with a benzaldehyde lyase and allowed the generation of chiral chroman-4-one derivatives with up to 98% ee.^226^

**Scheme 25:**
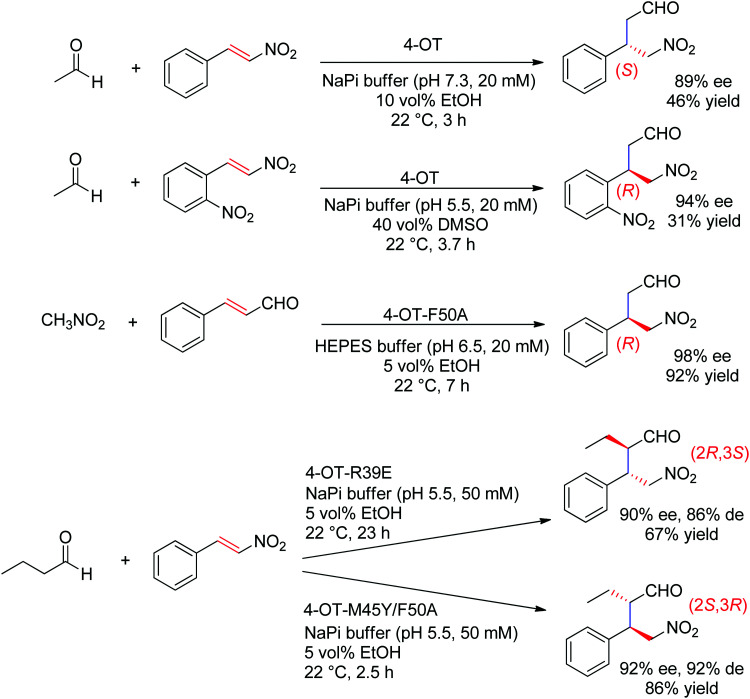
Examples of stereocomplementary strategies in 4-oxalocrotonate tautomerase (4-OT)-catalyzed Michael addition reactions.^195,227–229^

The chemically related Michael addition of acetaldehyde onto nitroalkenes was shown possible with 4-oxalocrotonate tautomerase (4-OT), an enzyme that relies on an N-terminal catalytic proline to activate the aldehyde (donor) substrate *via* enamine chemistry (Scheme 25). The products were obtained in high enantiopurity^227^ and the stereoselectivity could be controlled by substrate engineering, allowing access to both (*R*)- and (*S*)-γ-nitroaldehydes.^228^ Protein engineering also turned useful for the design of stereocomplementary biocatalysts.^229^ For donors other than acetaldehyde, a second chiral center was installed with high diastereoselectivity (up to 94% de). Finally, a smartly designed swap of functional groups along with a key mutation in 4-OT later allowed the reaction of nitroalkanes onto α,β-unsaturated aldehydes, and, in the case of nitromethane and cinnamaldehyde, elegantly provided stereocomplementary access to the γ-nitroaldehyde product (compared to the addition of acetaldehyde onto *trans*-β-nitrostyrene).^195^ Here, the unsaturated aldehyde acceptor was suggested to be activated by the proline *via* an iminium ion intermediate. Chemically analogous, a cupin loaded with copper was shown able to catalyze the asymmetric addition of nitromethane to azachalcones. Highest activity and stereoselectiviy were obtained after protein engineering and a single mutation was sufficient to obtain excellent values (up to 99% ee for the (*S*)-product with H52A).^230^

##### 1.3.2.2. Nonactivated alkenes

A few cases of enzymatic asymmetric C–C-bond forming reactions with nonactivated prochiral alkenes as electrophile have been reported and involve the catalytic promiscuity of enzymes that otherwise catalyze completely different chemical reactions.

Following the discovery of catalytic promiscuity of the cofactor independent ferulic acid decarboxylase (FDC) from *Enterobacter* sp. in asymmetric hydration reactions (see Section 1.2.2.2),^201^ a range of diverse nucleophiles was tested and demonstrated the ability of FDC to also form new C–N-, C–S- and C–C-bonds. The reaction with 4-hydroxystyrene was shown to proceed *via* asymmetric 1,6-conjugate addition of the nucleophile. The synthetically relevant addition of cyanide onto 4-vinylphenol delivered (*S*)-configurated 2-(4-hydroxyphenyl)propanenitrile in good yield and with 85% ee. Other phenolic acid decarboxylases were active, but despite higher stereoselectivity (up to 91% ee), conversions remained modest.^231^

A few creative C–C-bond forming protocols involving Old Yellow Enzymes have been developed, in which, following reduction by NAD(P)H, a key flavin radical semiquinone was obtained by photoexcitation. Under light irradiation, α-chloroamide derivatives underwent a reaction similar to the dehalogenation of α-bromoesters catalyzed by OYEs (see Section 2.1.3). The structure of the substrates was chosen such that cyclization occurred before the hydrogen atom transfer, thanks to a reactive C=C-bond within the molecule. A range of enantiopure lactams with diverse substitution pattern could be generated through this radical dehalogenation/stereoselective intramolecular C–C-bond forming protocol. Although up to two chiral centers could be formed, in that case diastereoselectivity was poor (<60% de).^232^

**Scheme 26:**
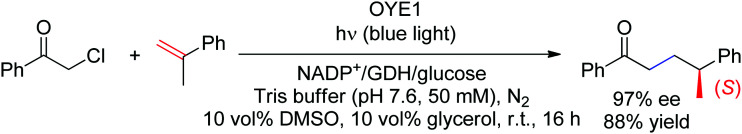
Asymmetric intermolecular hydroalkylation of nonactivated alkenes catalyzed by Old Yellow Enzyme under light irradiation (GDH: glucose dehydrogenase, OYE1: Old Yellow Enzyme from *Saccharomyces pastorianus*).^233^

More recently, a case of stereoselective formal intermolecular hydroalkylation of nonactivated alkenes catalyzed by Old Yellow Enzymes was reported by the Zhao lab (Scheme 26).^233^ In that case too, photoexcitation was crucial to initiate the radical reaction with α-halogenated carbonyl compounds. Overall, the reaction can be seen as the intermolecular equivalent of the cyclization developed with α-chloroamides (see above). The Hyster group later reported the same reaction with α-chloroamides devoid of C=C-double bonds, however stereoselectivity was on average less pronounced.^234^ In both cases, a γ-chiral center was generated and did not result from the radical C–C-bond forming step but from the final hydrogen atom transfer (HAT) from the flavin semiquinone.

Other asymmetric biocatalytic C–C-bond forming reactions exist, many derived from biosynthetic pathways. While these complementary methods are powerful approaches, they tend to be specific for particular substrate templates. They often involve a cyclization reaction (some are fully intramolecular) and will not be covered here.^205,235^

## 2. Enantioselective reactions

### 2.1. Conversion of prochiral carbons into chiral centers

#### 2.1.1. C–H hydroxylation

**Scheme 27:**
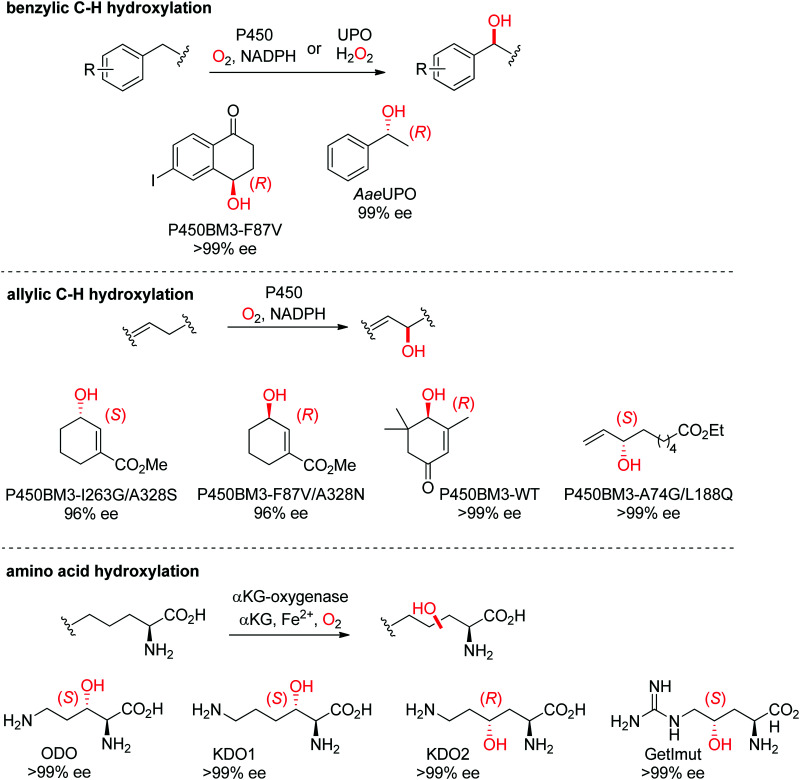
Range of hydroxylated products obtained by enantioselective C–H hydroxylation reactions catalyzed by cytochrome P450s, unspecific peroxygenases (UPOs) and α-ketoglutarate and nonheme iron-dependent oxygenases. α-KG: α-ketoglutarate, *Aae*UPO: UPO from *Agrocybe aegerita*,^237^ KDO1: l-lysine dioxygenase from *Catenulispora acidiphila*,^239^ KDO2: l-lysine dioxygenase from *Chitinophaga pinensis*,^239^ GetImut: engineered citrulline 4-hydroxylase from *Streptomyces* sp. L-49973,^241^ ODO: l-ornithine dioxygenase from *Catenulispora acidiphila*,^239^ P450BM3: P450 monooxygenase from *Bacillus megaterium*.^242,244–246^

Enantioselective hydroxylations *via* C–H activation can be accomplished by several enzymes (Scheme 27),^236^ the most notorious ones being P450 monooxygenases. Unspecific peroxygenases (UPO) are starting to compete in this area, in particular for benzylic hydroxylation reactions.^237,238^ Another option makes use of α-ketoglutarate and nonheme iron-dependent oxygenases, which have been especially employed for the regio- and enantioselective hydroxylation of amino acids.^239–241^ P450s were most of the times engineered to reach high enantioselectivity on a range of substrates, an approach which was particularly successful with the emblematic P450BM3 from *Bacillus megaterium*, both for benzylic^242,243^ and allylic C–H hydroxylations^244–246^ and for carbonyl α-hydroxylation.^247^ For hydroxylation of unactivated C–H bonds, some P450s and other systems (*e.g.*, peroxygenases) have shown good enantioselectivity.^248–250^ Finally, significant progresses have been achieved in the field of steroid hydroxylation. For instance, a P450 monooxygenase from *Streptomyces antibioticus* (OleP) could be engineered for the stereo- and regioselective functionalization of lithocholic acid toward ursodeoxycholic acid *via* a triple mutant. As with other challenging and poorly soluble substrates, conversions remained very low (max. 67 μM product from 2.5 mM substrate).^251^ The broader field of enzymatic hydroxylation of natural products, including that of terpene and macrolide scaffolds, has been recently reviewed.^248^

#### 2.1.2. Sulfoxidation

**Fig. 3:**
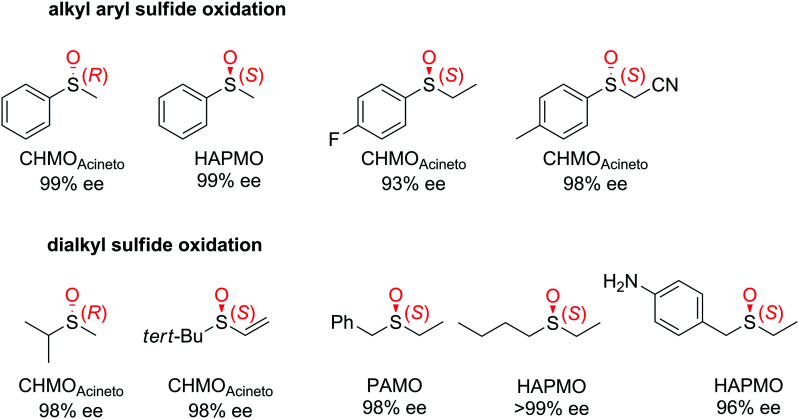
Range of chiral sulfoxides obtained by enantioselective sulfoxidation of alkyl aryl sulfides.^262–264^ CHMO_Acineto_: cyclohexanone monooxygenase from *Acinetobacter calcoaceticus* NCIMB 9871, HAPMO: 4-hydroxyacetophenone monooxygenase from *Pseudomonas putida* JD1, PAMO: phenylacetone monooxygenase from *Thermobifida fusca*. Reprinted from ref. 252 Copyright 2020, with permission from Elsevier.

The asymmetric oxidation of sulfides to chiral sulfoxides can be accomplished by monooxygenases, such as flavin-dependent Baeyer–Villiger monooxygenases (BVMOs), mostly known for their activity in the oxygenation of carbonyl compounds, and by flavin-containing monooxygenases (FMOs). In both cases, after activation of the flavin by reduction with NAD(P)H, further reaction with oxygen generates the reactive C(4a)-peroxyflavin. The latter gets then protonated to the hydroperoxyflavin intermediate, which can react as electrophile with soft nucleophiles, such as heteroatoms (S, N, P, Se). Relevant in asymmetric synthesis is the oxidation of thioethers to chiral sulfoxides.^252^ In that case, high chemoselectivity of the catalysts is important to not end up with over-oxidized nonchiral sulfone products. Other enzymes (*e.g.*, peroxidases, styrene monooxygenases, cytochrome P450 monooxygenases and dioxygenases)^253^ can oxidize sulfides, however, from a practical synthetic standpoint, BVMOs are superior in terms of yields and enantioselectivity,^254^ and are usually employed for small substrates with a diverse substitution pattern. Alkyl aryl^262–264^ and dialkyl sulfides^258–261^ are converted to the corresponding sulfoxides with excellent ee values (Fig. 3). Owing to the large protein diversity of BVMOs, enantiocomplementary enzymes on defined substrates allow access to products in both absolute configurations, as seen in the oxidation of methyl phenyl sulfide (Fig. 3).

**Scheme 28:**
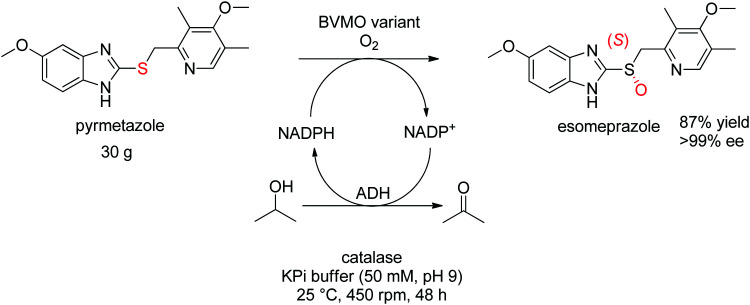
Asymmetric sulfoxidation of pyrmetazole to (*S*)-esomeprazole using an engineered Baeyer–Villiger monooxygenase (BVMO variant).^265^ Adapted with permission from ref. 265. Copyright 2018 American Chemical Society.

For more challenging substrates, directed evolution was successfully applied. An engineered BVMO developed by Codexis was employed in the asymmetric oxidation of pyrmetazole, a precursor to the drug esomeprazole used in the treatment of stomach acid reflux. The (*S*)-enantiomer of the target product could be obtained in 87% yield and in enantiopure form (>99% ee) from a reaction run on 30 g scale. The coupled-enzyme approach was selected for the regeneration of NADPH, and relied on isopropanol as reductant and an alcohol dehydrogenase (Scheme 28).

#### 2.1.3. (De)halogenation

**Scheme 29:**
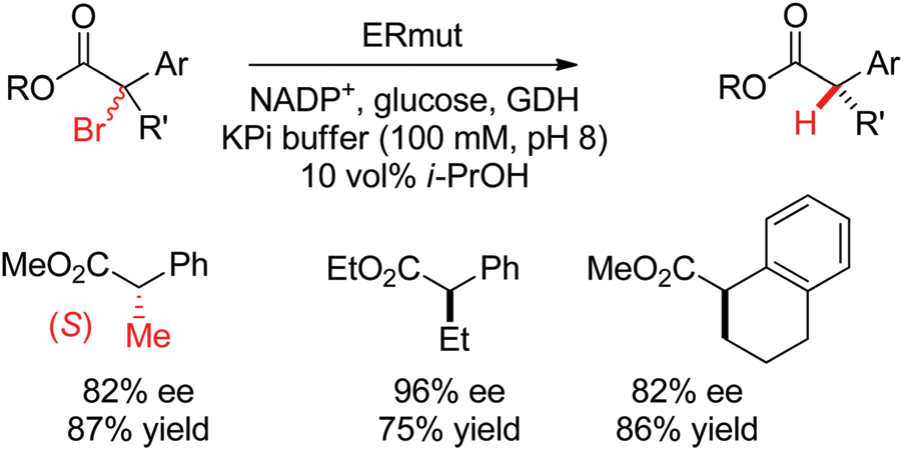
Enantioselective dehalogenation of α-bromoesters by a tyrosine mutant of ene-reductase from *Gluconobacter oxydans* (ERmut).^266^ GDH: glucose dehydrogenase.

Ene-reductases from the Old Yellow Enzyme family have been employed in a variety of enantioselective reactions beyond stereoselective reduction reactions (see Section 1.1.1). The capacity of the reduced flavin to react *via* a radical mechanism with halogenated substrates was exploited for the enantioselective dehalogenation of α-bromoesters. In the course of the reaction, electron transfer from the flavin to the substrate was associated with loss of bromide. The thereby generated flavin semiquinone subsequently transferred a hydrogen to the radical intermediate in an enantioselective manner and led to the formation of the final chiral esters with high ee values (up to 96% ee).^266^ Since the substrate was first transformed into a radical species, the chiral information at the pre-existing α-center was not relevant. The system was applied to a wide variety of substrates and relied on the glucose/glucose dehydrogenase system to regenerate NADPH necessary for the first reductive event (Scheme 29).

Despite recent and significant advances in the identification of enzymes active in halogenation reactions,^267,268^ the field of biocatalytic asymmetric halogenation remains highly underdeveloped.^269^ Recently, this area gathered particular attention as cases of enantioselective biohalogenation were reported.^270,271^ Most notably, the potential in biocatalysis of WelO5, a carrier-independent iron/α-ketoglutarate dependent halogenase reported to chlorinate regio- and enantioselectively 12-epi-fischerindole U, has been explored.^272^ Engineering of a highly similar homologue, WelO5*, led to variants with an enlarged substrate scope, albeit with low catalytic activity.^273^ In parallel, complementary engineering efforts on a homologous protein from *Westiella intricata* HT-29-1 by structure-guided directed evolution delivered enzymes with excellent chemo-, regio-, and diastereoselective chlorination activity on hapalindole derivatives.^274^ While promising, these enzymes will have to demonstrate generality to become potent tools in synthesis on a broad panel of nonnatural substrates at synthetically relevant concentration.^275,276^ In contrast, for the halogenation of aromatic molecules, several enzymatic protocols are available.^269^

### 2.2. (Dynamic) kinetic resolution

In cases where the chiral center is already in place in the starting material, the enantioselective transformation of racemic molecules into enantiopure compounds can be attained by several strategies that rely on enzymatic kinetic resolution.^277^ The underlying concept is that a biocatalyst, facing two enantiomers of the same substrate, will preferentially convert one enantiomer to the final product. In an ideal case, the reaction with the other enantiomer is so slow that it will not be observed on the timescale of the reaction and the final product is therefore obtained with high enantiopurity in a maximum 50% yield. 50% of the starting material is recovered in enantiopure form. When an *in situ* racemization step specific for the substrate is included, a maximum product yield of 100% can be reached since the nonreacting enantiomer is transformed through racemization into the preferred enantiomer that reacts with the biocatalyst of interest (dynamic kinetic resolution). The final product is obtained with high enantiopurity. Historical examples with lipases will not be treated here and the reader can consult the abundant literature for more information and examples.^16–18,208,278,279^ Instead, the focus will be briefly directed to recent examples of kinetic resolution and dynamic kinetic resolution with redox reactions, such as those involving α-substituted carbonyl compounds, which usually racemize spontaneously in (slightly basic) aqueous media.

**Scheme 30:**
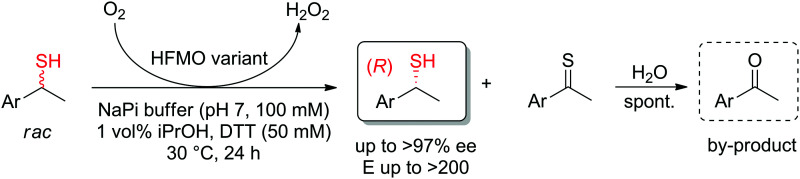
Kinetic resolution of *sec*-thiols through enantioselective oxidation catalyzed by variants of 5-(hydroxymethyl)furfural oxidase (HFMO).^280^

The kinetic resolution of secondary thiols was achieved by enantioselective oxidation using an engineered alcohol oxidase. While the wild-type enzyme was inactive, several variants of the enzyme HFMO (5-(hydroxymethyl)furfural oxidase) could catalyze the oxidation of (*S*)-1-arylethanethiols and in several cases, perfect kinetic resolution was obtained (*E* > 200), with conversions reaching max. theoretical yield of 50%. Enantiopure secondary (*R*)-thiols were obtained, along with acetophenone derivatives, which formed spontaneously by hydrolysis of the oxidized thioketone products (Scheme 30).^280^

The light-driven kinetic resolution of α-amino and α-hydroxy carboxylic acids was made possible through (*S*)-selective decarboxylation catalyzed by the fatty acid photodecarboxylase from *Chlorella variabilis* NC64A (*Cv*FAP). *E* values up to >200 could be achieved and highest turnover numbers (TON) were obtained with the single variant *Cv*FAP-G462Y (up to ∼4000). The reaction conducted under irradiation at 450 nm at 20 °C was scaled up to 100 mg and allowed the isolation of (*R*)-2-hydroxyoctanoic acid in 45% yield and 99% ee, and of (*R*)-2-hydroxyhexanoic acid in 40% yield and 98% ee.^281^

**Scheme 31:**
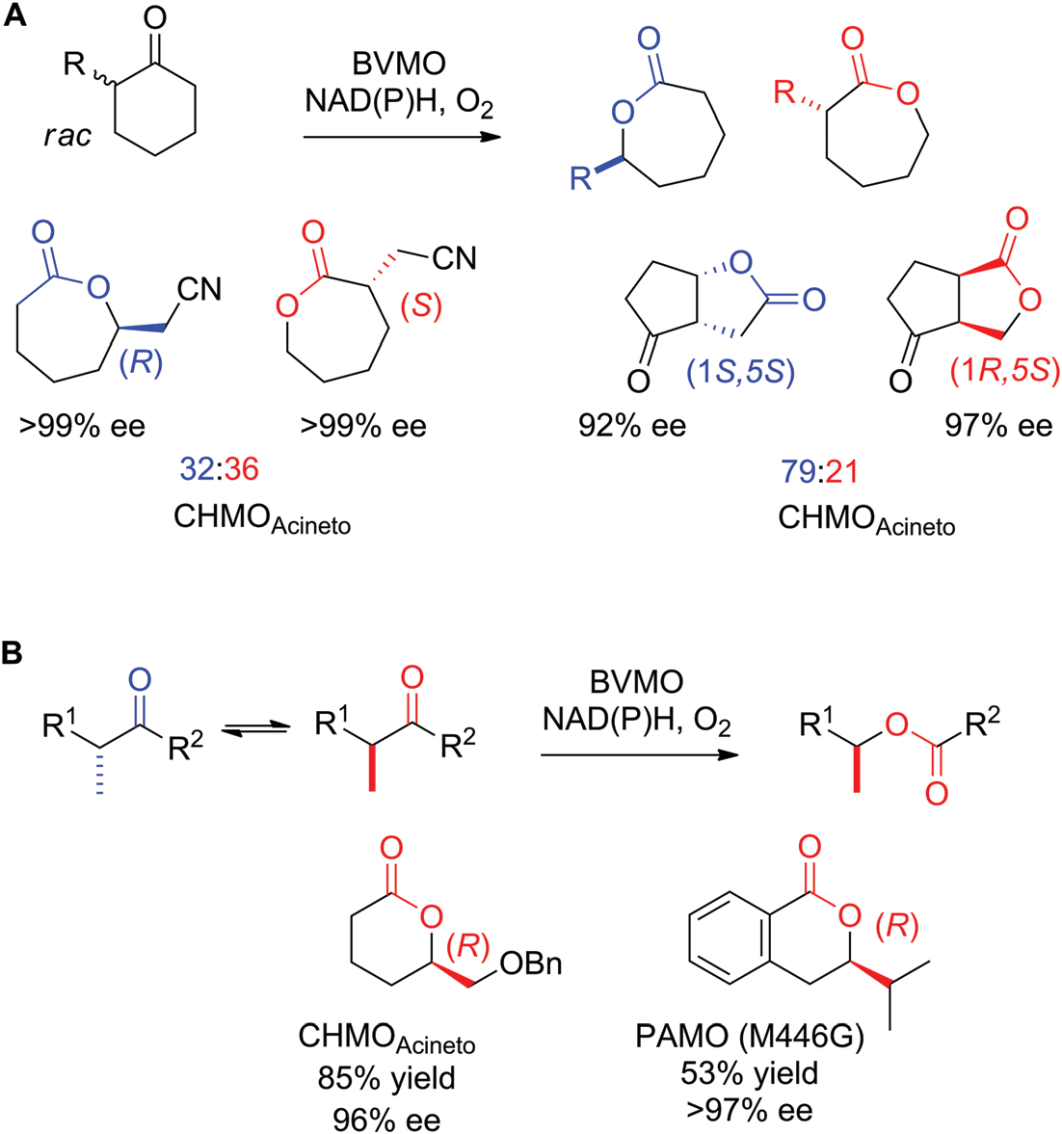
Applications of enantioselective Baeyer–Villiger monooxygenases in (A) enantiocomplementary regiodivergent process^283,284^ and (B) dynamic kinetic resolution of racemic substrates^285–287^ with examples of products obtained with high enantiopurity. CHMO_Acineto_: cyclohexanone monooxygenase from *Acinetobacter calcoaceticus* NCIMB 9871, PAMO (M446G): variant of phenylacetone monooxygenase from *Thermobifida fusca.* Reprinted from ref. 252 Copyright 2020, with permission from Elsevier.

Enantioselective Baeyer–Villiger monooxygenases (BVMOs) have been employed in the kinetic resolution of racemic α-substituted ketones and lactones.^252,277^ Owing to exquisite selectivity, *E* values >200 have been obtained with 2-substituted cyclopentanone and cyclohexanone derivatives, as well as with (aryl) aliphatic ketones.^282^ With particular compounds, cases of enantiocomplementary regiodivergent monooxygenation have been observed, in which the two enantiomers of a chiral substrate were converted to two regioisomeric products with high enantiopurity (Scheme 31A).^283,284^ The application of BVMOs under basic pH (9–10)^285^ or in combination with a weekly basic anionic exchange resin^286^ promoted the dynamic kinetic resolution of α-alkylated ketones through *in situ* racemization of the substrates (Scheme 31B).^287^

Highly stereo- and enantioselective ADHs haven been employed in dynamic kinetic resolution processes of α-substituted chiral carbonyl compounds toward formation of optically pure α-alkyl-β-hydroxy esters,^288^ α-alkyl-β-hydroxy ketones, α-amino-β-hydroxy esters, and variously substituted halohydrins.^82,289,290^ The stereoselective reduction reaction combined with the stereorecognition of the existing chiral center and *in situ* racemization of the substrate ensure that the final products possessing two chiral centers are obtained as one stereoisomer only of the four theoretically possible ones in high yields.

**Scheme 32:**
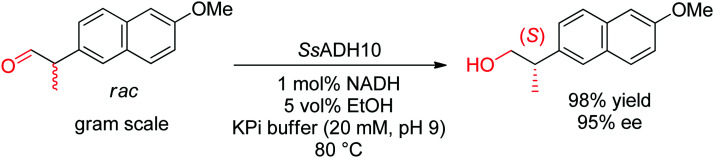
Reductive dynamic kinetic resolution of α-substituted aldehyde catalyzed by an alcohol dehydrogenase from *Sulfolobus sulfataricus* (*Ss*ADH10) to access precursor of (*S*)-naproxen.^291^ Republished from ref. 38 with permission of John Wiley & Sons – Books, copyright 2021; permission conveyed through Copyright Clearance Center, Inc.

With α-substituted aldehydes, similar approach is possible and leads to enantiopure α-substituted primary alcohols. (*S*)-Profenols could be obtained in excellent enantiopurity (up to 99% ee) by applying a thermostable ADH from *Sulfolobus sulfataricus* to the enantioselective reduction of a series of racemic 2-arylpropionaldehydes at pH 9, including the precursor to naproxen (Scheme 32).^291^ The reduced nicotinamide was regenerated by the coupled-substrate approach employing ethanol as hydride source.

**Scheme 33:**
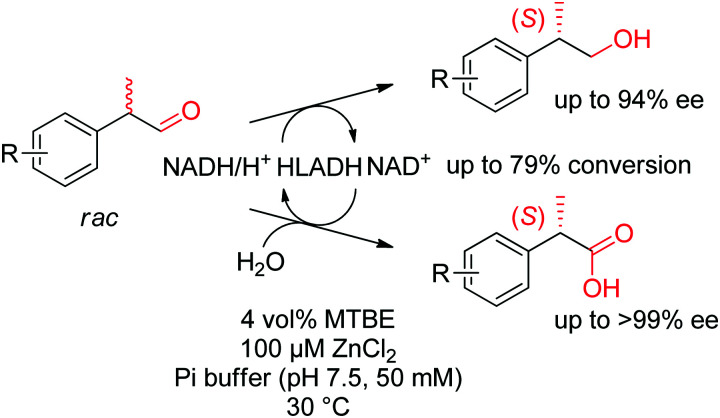
Asymmetric disproportionation of racemic 2-arylpropanals catalyzed by alcohol dehydrogenase from horse liver (HLADH) under redox-neutral conditions to form (*S*)-profens and profenols.^292^ Reproduced from ref. 129 with permission from the Royal Society of Chemistry, copyright 2019.

Under different reaction conditions, simultaneous access to (*S*)-profens and profenols was granted following the disproportionation of racemic 2-arylpropanals catalyzed by ADH from horse liver (HLADH). This parallel interconnected dynamic asymmetric transformation delivered a 1 : 1 mixture of corresponding alcohols and carboxylic acids, which were obtained with high enantiopurity (up to 99% ee) and high conversion levels (Scheme 33).^292^ Racemization of the substrate was observed at the reaction pH of 7.5. The system rendered a redox-neutral cascade and only catalytic amounts of the oxidized nicotinamide cofactor were necessary.

**Scheme 34:**
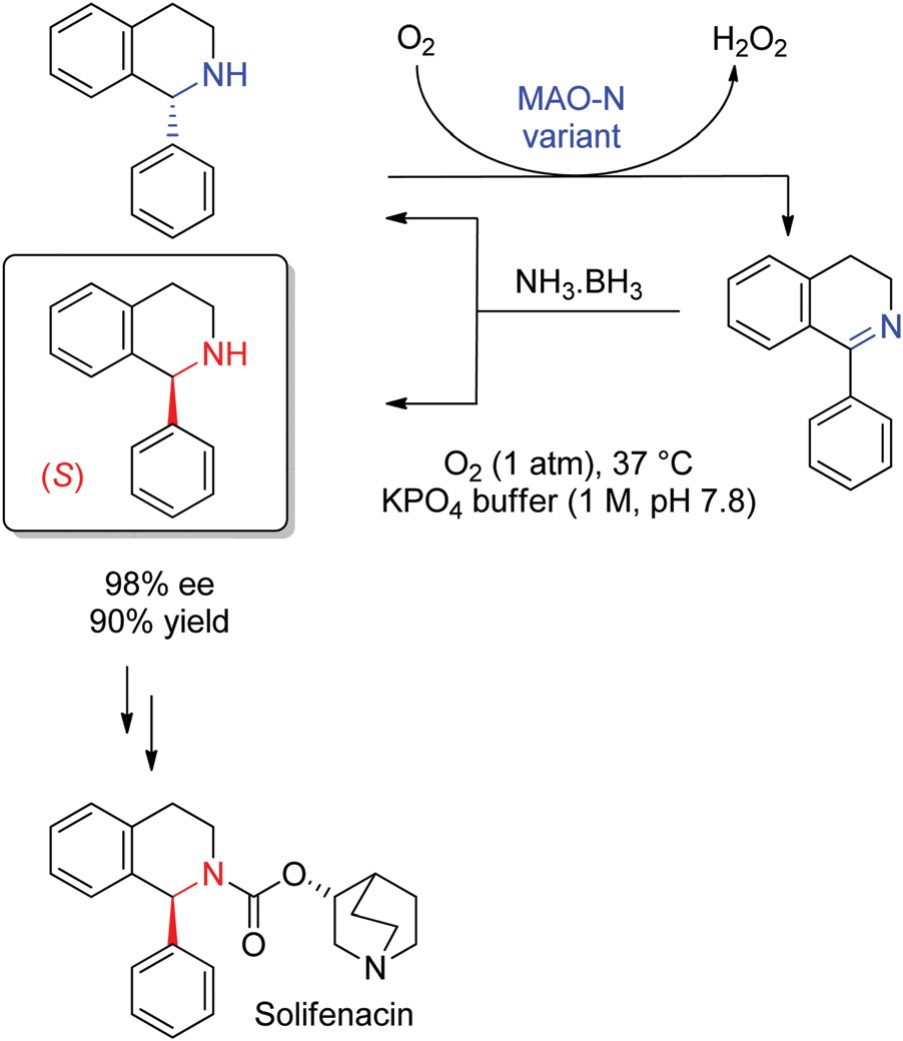
Cyclic deracemization of *rac*-1-phenyl-1,2,3,4-tetrahydroisoquinoline through enantioselective oxidation by a variant of monoamine oxidase from *Aspergillus niger* (MAO-N) and nonselective reduction with ammonia–borane complex. The (*S*)-amine was then employed to access solifenacin in two steps.^295^

Complementary to the numerous enzymatic methods available to generate chiral amines (see Sections 1.1.3 and 1.2.1), kinetic resolution of racemic amines *via* oxidative protocols offers very efficient approaches that usually rely on flavin-dependent amine oxidases,^293,294^ in particular amino acid oxidases and most prominent monoamine oxidases (MAOs). From the latter class of enzymes, MAO from *Aspergillus niger* (MAO-N) has been employed in a wide range of deracemization protocols, due to its broad substrate scope and acceptance of primary and secondary amines. A large number of variants have been generated and allow the oxidation of many substrates, including tertiary amines.^295,296^ A ‘simple’ kinetic resolution leads in the case of perfect enantioselectivity to maximum 50% yield of the nonconverted enantiopure amine and generates 50% side product in form of an imine (or iminium ion) that usually hydrolyzes to the ketone. Combining (non)selective (chemical) reduction of the imine back to the (racemic) amine with the enzymatic oxidation step leads to a cyclic deracemization in which yields may reach 100% for the enantiopure desired chiral amine. A variant of MAO-N was identified during a directed evolution campaign that showed strong preference for the oxidation of (*S*)-α-methylbenzylamine. Combined with a nonselective reduction using the ammonia–borane complex, (*R*)-α-methylbenzylamine could be obtained with 93% ee and 77% yield.^297^ This process was later applied to the deracemization of key intermediates to active pharmaceutical ingredients. In the case of 1-phenyl-1,2,3,4-tetrahydroisoquinoline, a precursor to solifenacin used in the treatment of overactive bladder, the (*S*)-enantiomer could be obtained in 90% isolated yield and 98% ee after 48 h reaction time at the gram-scale (Scheme 34).^295^ The wild-type and variants of MAO-N have been applied in a number of synthetic sequences,^298^ either multi-enzymatic, such as for the synthesis of chiral 2,5-disubstituted pyrrolidines triggered by a transaminase-catalyzed step,^299^ or chemoenzymatic, as seen in the synthesis of a chiral precursor to boceprevir, a drug developed by Merck & Co, Inc. for the treatment of hepatitis C.^300^

## 3. Atroposelective reactions

Enzymatic protocols used in asymmetric synthesis are predominantly applied to the generation of central chirality, mostly tetrasubstituted carbon atoms, occasionally chiral sulfur atoms (see Section 2.1.2). Nevertheless, stereoselective enzymatic strategies also exist to generate products with axial chirality in enantiopure form. Cases in which planar chirality is involved will not be discussed here. For larger sampling of existing examples dealing with noncentral chirality with enzymes, the reader is referred to excellent reviews and articles broadly covering the topic.^301–305^

### 3.1. (Dynamic) kinetic resolution of axially chiral molecules

Initial studies dedicated to asymmetric biotransformations involving noncentral chirality were focusing on reactions catalyzed by hydrolytic enzymes. Those were employed in the kinetic resolution of racemic axially chiral molecules in both reaction directions (hydrolysis of esters and transesterification reaction). For instance, pig liver esterase was found to hydrolyze racemic allenic esters with some enantioselectivity (*E* value up to 42), leading to formation of the corresponding acids in 83% ee,^306,307^ while lipases were used for the acylation of primary allenic alcohols by vinyl butyrate and vinyl acetate. Excellent enantioselectivities were obtained with a few substrates under perfect kinetic resolution (*E* value >200).^308,309^ Later, that system could be upgraded to a dynamic kinetic resolution with the introduction of a palladium-catalyzed isomerization of the substrate that was compatible with the biocatalyzed acylation and led to the formation of optically active allenes in up to 83% yield and 89% ee.^310^

**Scheme 35:**
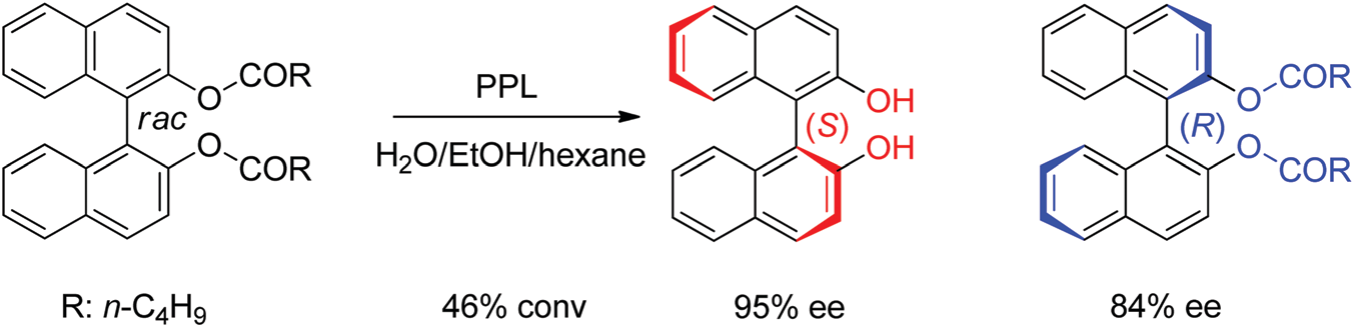
Kinetic resolution of racemic 1,1′-binaphthyl-2,2′-diol based diesters through atroposelective hydrolysis to corresponding diols catalyzed by porcine pancreatic lipase (PPL).^311^

Such biocatalytic hydrolytic kinetic resolutions have been successfully applied to atropisomeric biaryl compounds. Several enzyme preparations (porcine pancreatic lipase and cholesterol esterase for instance) could efficiently catalyze the atroposelective hydrolysis of racemic 1,1′-binaphthyl-2,2′-diol based diesters, leading to the formation of the diol with excellent ee value and *E* value up to 105 (Scheme 35).^311,312^ Lipase-catalyzed monoacylation reactions were also developed for binaphthol and proceeded with high stereoselectivity.^313^

Recently, a dynamic kinetic resolution of racemic axially chiral 2,2′-dihydroxy-1,1′-biaryls was achieved by including to the biocatalytic monoacylation step an *in situ* racemization by a Ru(ii)-based catalyst. Several biaryl diols could be obtained in high yields and with up to 98% ee after final base-mediated deacylation.^314^

**Scheme 36:**
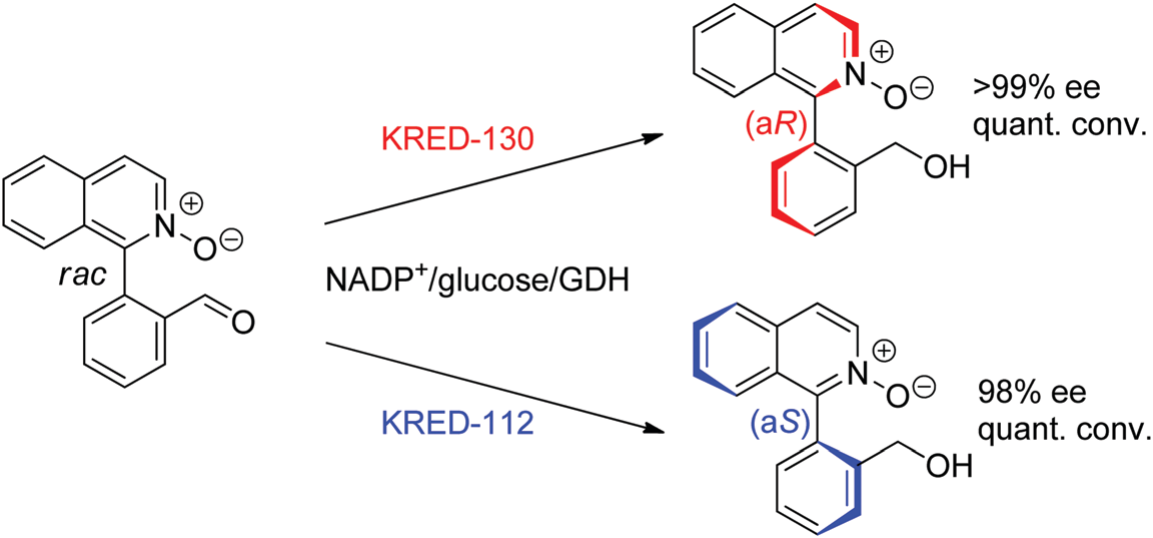
Dynamic kinetic resolution of configurationally unstable substrates *via* atroposelective reduction by commercial alcohol dehydrogenases (KRED-130 and KRED-112) toward formation of atropisomeric biaryl *N*-oxides (GDH: glucose dehydrogenase).^318^

Importantly, although still underexplored, atroposelective kinetic resolutions have also been established *via* enzymatic redox chemistry. Early studies reported the microbial full oxidation of α-allenic alcohols to their corresponding acids, however obtained ee values remained modest.^315,316^ In one of the first reported examples with biaryl compounds, baker's yeast was applied to the reduction of non *C*_2_-symmetric substrates such as 2-formyl-1,1′-binaphthyls, here again the ee values of the product did not exceed 70%.^317^ More recently, perfect enantioselectivity could be obtained by employing commercial ADHs in the reduction of biaryl monoaldehydes displaying additional *N*-oxide functionalization. When applied to configurationally unstable substrates, an elegant dynamic kinetic resolution could be obtained. This was possible due to the more stable chiral axis in the resulting product, which was finally obtained in quantitative yield and >95% ee in a stereocomplementary manner with enzymes of opposite selectivity (Scheme 36).^318^ The products were employed as Lewis base organocatalysts in asymmetric allylation of aldehydes.

### 3.2. Conversion of prochiral compounds into axially chiral compounds

Axially chiral compounds exist in enantiopure form in nature (*e.g.*, gossypol, vancomycin). Their synthesis often involves oxidative coupling reactions catalyzed by cytochrome P450 enzymes or laccases.^319^ A few studies have shown the successful coupling of two units *via* enzymatic phenol coupling reactions using laccases.^320,321^ Atroposelective behavior could be confirmed with several fungal laccases in the regioselective dimerization of γ-naphthopyrone to both atropisomers of ustilaginoidin A.^322^ Other stereoselective dimerization systems based on laccases and dirigent proteins^323–326^ and P450s^327–329^ are emerging. Though promising, such approaches are currently limited to the synthesis of natural product derivatives and will necessitate further improvements to be employed with nonnatural molecules in broadly applicable synthetic routes.

## Outlook

Biocatalysis could be established over the past two decades as a powerful methodology in asymmetric synthesis mostly owing to its interdisciplinary character. Many of the examples treated in this review highlight the advantages of merging biocatalysis with methodologies of biotechnology, molecular biology, protein engineering, bioinformatics, or synthetic biology, to name a few complementary areas. Several waves of technological development have been recognized by experts over the last decade.^330,331^ Writing 2021, a new revolution – more than a wave – triggered by artificial intelligence (AI) is already on the rise. The recent spectacular example of AlphaFold from DeepMind in accurately predicting the structure of a protein from its amino acid sequence will certainly become disruptive to the whole field of protein science.^332,333^ Computational tools have long contributed to revolutionize the field of biocatalysis, including protein engineering campaign, modelling and docking, and mechanistic investigations. In particular in asymmetric synthesis, the accurate prediction of protein 3D structures, combined with other methodologies such as molecular docking and protein engineering, will become big assets in speeding up the development of robust and selective biocatalysts to generate a broad range of enantiopure molecules useful in the fine chemical and pharmaceutical sector.^334^

In addition to the increasing availability and diversity of commercial enzymes in form of off-the-shelf or customized biocatalysts,^38^ other aspects are contributing to rendering enzymes accessible to chemists willing to incorporate these catalysts in their synthesis planning. Retrobiosynthesis for instance is an emerging area that provides keys to apply biosynthetic logic to the design of artificial synthesis routes.^335,336^ While mimicking the retrosynthetic approach developed by Corey,^337,338^ the focus remains on key enzymatic steps to inspire retrobiocatalytic strategies.^339,340^ Biocatalysis offers many ways to access key functional group interconversions and computational tools are now available^341–343^ to help identify disconnections – a key strategy in retrosynthesis – and suitable starting materials with corresponding enzymes toward a target molecule.

The field of catalysis in general has revolutionized how (chiral) molecules were made during the 20th century. Being able to combine the advantages of the many disciplines in catalysis (metal, organo-, bio, photo-catalysis) in cascade processes^344^ will define how chemicals of the 21st century are made.^345–348^ Several examples are paving the way toward the merging of enzymes with chemical methods in elaborated (cascade) synthesis.^349–353^

Finally, the growing importance of (meta)genomics and ultrahigh-throughput screening, such as seen with microfluidic approaches,^354^ will raise new challenges. Generating large data set from vast and diverse protein libraries is not technically limited anymore, instead the interpretation of the rapidly growing amount of information remains currently an obstacle to advancing further and faster. Beyond helping in solving the ‘protein folding problem’, AI is mature to tackle the big data problem emerging in biocatalysis. Game changer to the field of asymmetric biocatalysis will be the successful prediction of enzyme structure–activity–stereoselectivity pattern. While this is already possible for some classes of enzymes, and has led to the establishment of popular rules (the ‘Prelog’ rule with alcohol dehydrogenases^86^ or the ‘Kazlauskas’ rule with esterases and lipases^355^), many more enzyme classes still rely on large screening efforts to identify the ideal substrate-enzyme pair for the generation of the targeted product, despite mechanistic understanding at the molecular level.

As witnessed in the currently challenging pandemic situation worldwide, accessing biologically active molecules quickly has become vital and the merging of all the above-mentioned disciplines will be essential to making an impact in this area. The recent example of the multi-step fully biocatalytic synthesis of islatravir, a potential drug for treatment of HIV developed by Merck & Co, Inc.,^256^ indicates that the future of biocatalysis in the field of asymmetric synthesis is bright,^255,257^ in particular in the context of a multi-disciplinary science.

## Conflicts of interest

There are no conflicts to declare.

## Supplementary Material
